# Supplementary material for: Bayesian Workflow for Generative Modeling in Computational Psychiatry
Source: Comput Psychiatr. 2025 Mar 25;9(1):76–99. doi: 10.5334/cpsy.116 (PMC11951975; doi:10.5334/cpsy.116)
Supplement: Supplementary Material. — Supplementary Figures and Analyses. [file cpsy-9-1-116-s1.pdf]

Supplementary Material

Bayesian Workflow for Generative Modeling in Computational Psychiatry

Alexander J. Hess, Sandra Iglesias, Laura Köchli, Stephanie Marino, Matthias Müller-Schrader, Lionel Rigoux, Christoph Mathys, Olivia K. Harrison, Jakob Heinzle, Stefan Frässle, Klaas Enno Stephan

S1. Examples of individual participants' behavioural data and fits of M1 (main data set)

Figures S1A-S1D show behavioural data and model fits (M1) of the four participants for which M1 fit the data best as measured by the log likelihood.

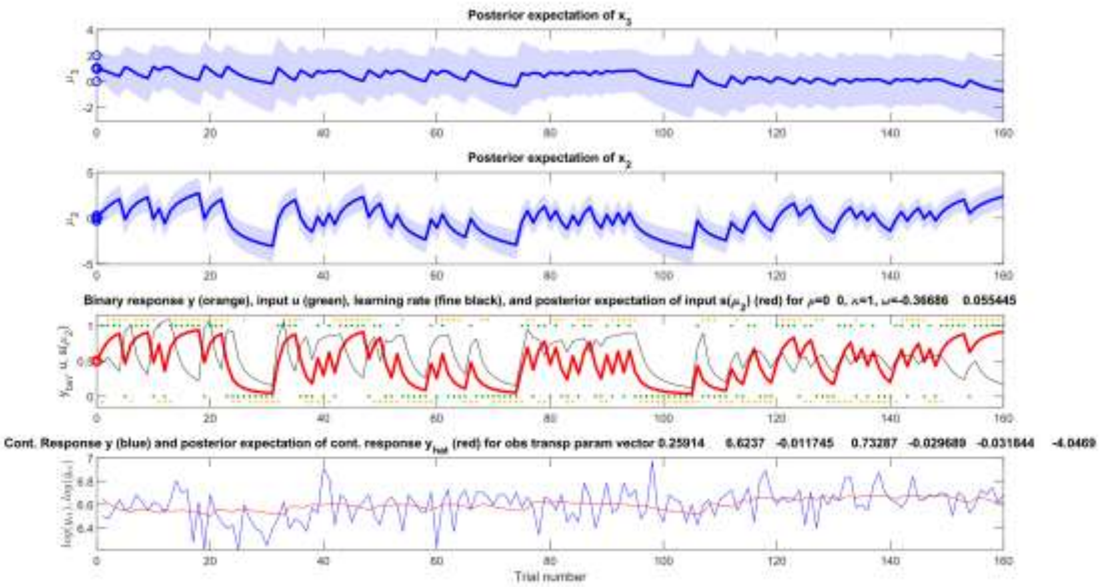

Figure S1A | Behavioural data and model fits (M1) of participant 45 of the main data set. The top panel shows the fitted mean belief μ_3 about the log volatility of the environment in blue with the blue shaded area representing the uncertainty about x_3, i.e., the variance σ_3. The second panel shows mean μ_2 and variance σ_2 of the belief about the cue-outcome contingency. The second lowest panel shows in red the mean belief about the outcome $\hat{\mu}_1$ given one of the two fractals, the actual outcomes for one of the two fractals as green dots (1 = reward, 0 = no reward) and which of the two fractals was selected as yellow dots. The fine black line represents an implied learning rate at the level of the outcome calculated as $\mathbb{1}_{\{\delta_1 \neq 0\}} \left(\frac{\Delta s(\mu_2)}{u - \Delta s(\mu_2)} \right)$ where s is the sigmoid function. The lowest panel shows the empirical log RTs [ms] as blue line and the fitted log RT trajectory in red.

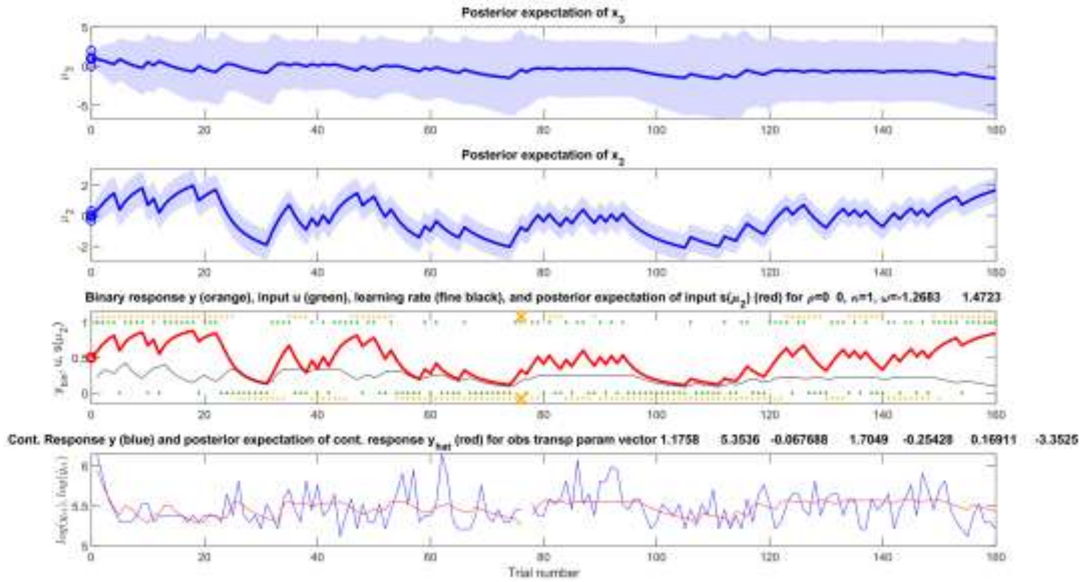

Figure S1B | Behavioural data and model fits (M1) of participant 3 of the main data set. The top panel shows the fitted mean belief μ_3 about the log volatility of the environment in blue with the blue shaded area representing the uncertainty about x_3, i.e., the variance σ_3. The second panel shows mean μ_2 and variance σ_2 of the belief about the cue-outcome contingency. The second lowest panel shows in red the mean belief about the outcome $\hat{\mu}_1$ given one of the two fractals, the actual outcomes for one of the two fractals as green dots (1 = reward, 0 = no reward) and which of the two fractals was selected as yellow dots. The fine black line represents an implied learning rate at the level of the outcome calculated as $\mathbb{1}_{\{\delta_1 \neq 0\}} \left(\frac{\Delta s(\mu_2)}{u - \Delta s(\mu_2)} \right)$ where s is the sigmoid function. The lowest panel shows the empirical log RTs [ms] as blue line and the fitted log RT trajectory in red.

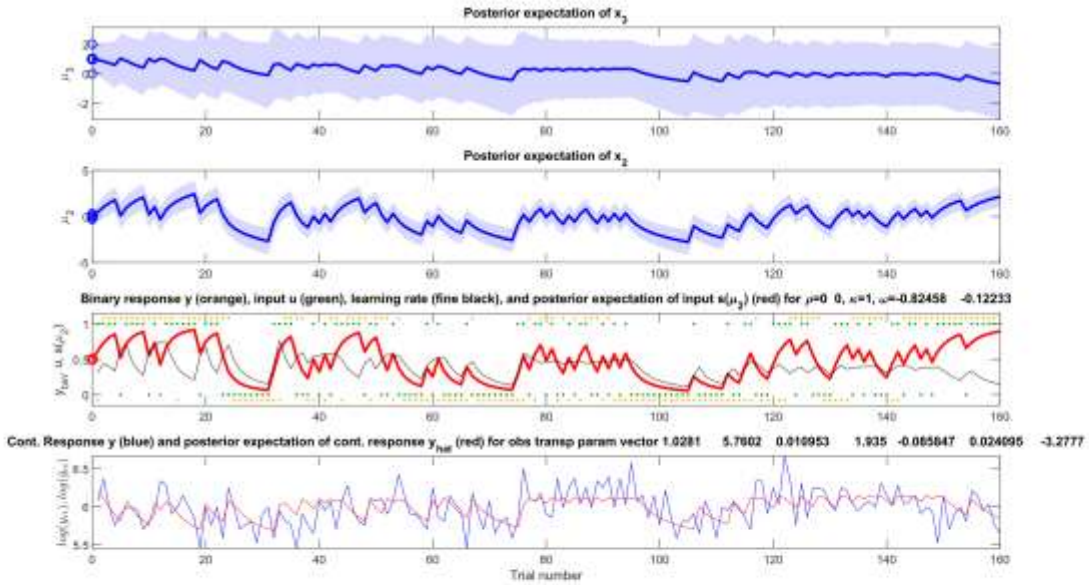

Figure S1C | Behavioural data and model fits (M1) of participant 21 of the main data set. The top panel shows the fitted mean belief μ_3 about the log volatility of the environment in blue with the

blue shaded area representing the uncertainty about x_3, i.e., the variance σ_3. The second panel shows mean μ_2 and variance σ_2 of the belief about the cue-outcome contingency. The second lowest panel shows in red the mean belief about the outcome $\hat{\mu}_1$ given one of the two fractals, the actual outcomes for one of the two fractals as green dots (1 = reward, 0 = no reward) and which of the two fractals was selected as yellow dots. The fine black line represents an implied learning rate at the level of the outcome calculated as $\mathbb{1}_{\{\delta_1 \neq 0\}} \left(\frac{\Delta s(\mu_2)}{u - \Delta s(\mu_2)} \right)$ where s is the sigmoid function. The lowest panel shows the empirical log RTs [ms] as blue line and the fitted log RT trajectory in red.

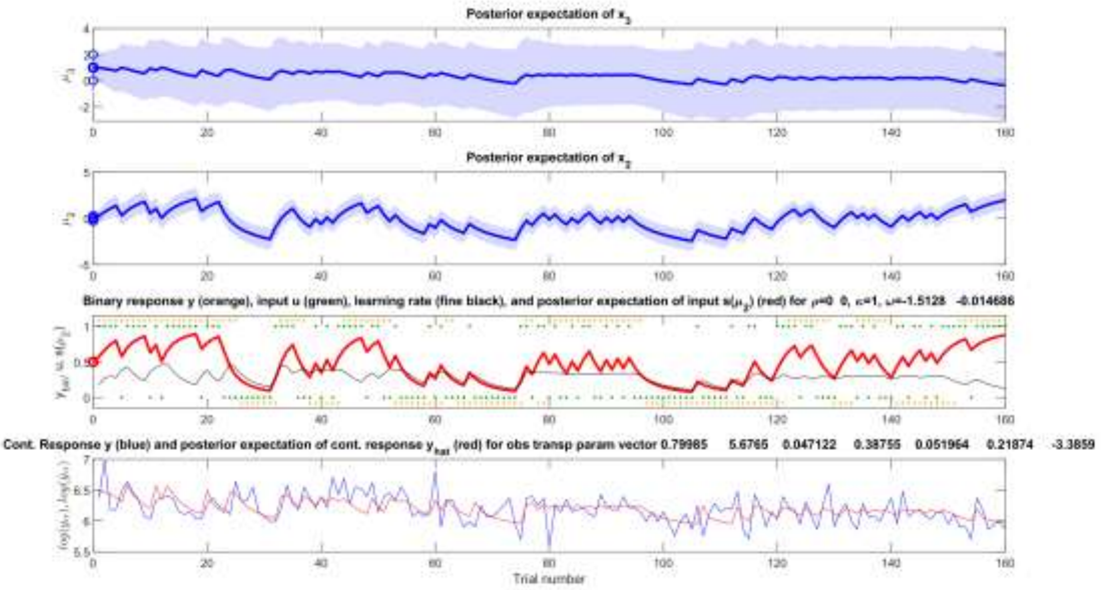

Figure S1D | Behavioural data and model fits (M1) of participant 33 of the main data set. The top panel shows the fitted mean belief μ_3 about the log volatility of the environment in blue with the blue shaded area representing the uncertainty about x_3, i.e., the variance σ_3. The second panel shows mean μ_2 and variance σ_2 of the belief about the cue-outcome contingency. The second lowest panel shows in red the mean belief about the outcome $\hat{\mu}_1$ given one of the two fractals, the actual outcomes for one of the two fractals as green dots (1 = reward, 0 = no reward) and which of the two fractals was selected as yellow dots. The fine black line represents an implied learning rate at the level of the outcome calculated as $\mathbb{1}_{\{\delta_1 \neq 0\}} \left(\frac{\Delta s(\mu_2)}{u - \Delta s(\mu_2)} \right)$ where s is the sigmoid function. The lowest panel shows the empirical log RTs [ms] as blue line and the fitted log RT trajectory in red.

Figures S1E and S1F show behavioural data and model fits (M1) of two participants that show an average fit as measured by the log likelihood.

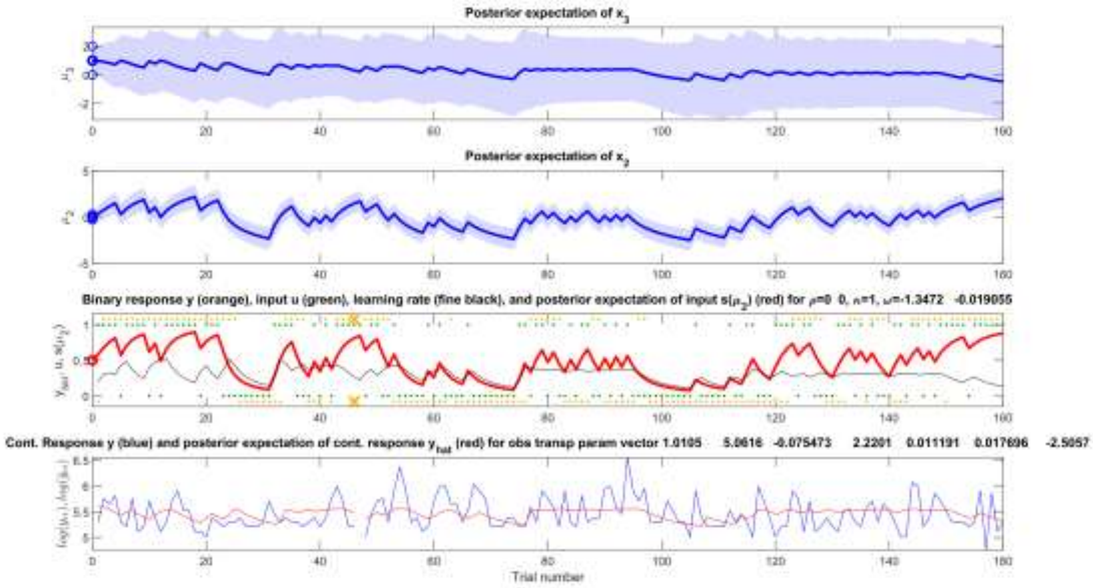

Figure S1E | Behavioural data and model fits (M1) of participant 28 of the main data set. The top panel shows the fitted mean belief μ_3 about the log volatility of the environment in blue with the blue shaded area representing the uncertainty about x_3, i.e., the variance σ_3. The second panel shows mean μ_2 and variance σ_2 of the belief about the cue-outcome contingency. The second lowest panel shows in red the mean belief about the outcome $\hat{\mu}_1$ given one of the two fractals, the actual outcomes for one of the two fractals as green dots (1 = reward, 0 = no reward) and which of the two fractals was selected as yellow dots. The fine black line represents an implied learning rate at the level of the outcome calculated as $\mathbb{1}_{\{\delta_1 \neq 0\}} \left(\frac{\Delta s(\mu_2)}{u - \Delta s(\mu_2)} \right)$ where s is the sigmoid function. The lowest panel shows the empirical log RTs [ms] as blue line and the fitted log RT trajectory in red.

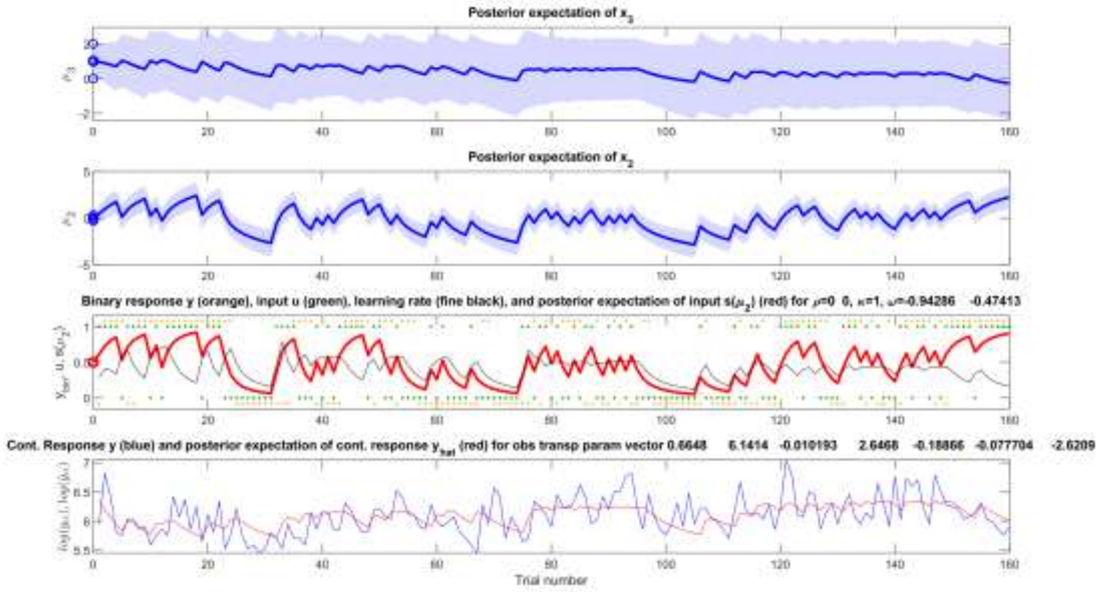

Figure S1F | Behavioural data and model fits (M1) of participant 29 of the main data set. The top panel shows the fitted mean belief μ_3 about the log volatility of the environment in blue with the

blue shaded area representing the uncertainty about x_3, i.e., the variance σ_3. The second panel shows mean μ_2 and variance σ_2 of the belief about the cue-outcome contingency. The second lowest panel shows in red the mean belief about the outcome $\hat{\mu}_1$ given one of the two fractals, the actual outcomes for one of the two fractals as green dots (1 = reward, 0 = no reward) and which of the two fractals was selected as yellow dots. The fine black line represents an implied learning rate at the level of the outcome calculated as $\mathbb{1}_{\{\delta_1 \neq 0\}}\left(\frac{\Delta s(\mu_2)}{u - \Delta s(\mu_2)}\right)$ where s is the sigmoid function. The lowest panel shows the empirical log RTs [ms] as blue line and the fitted log RT trajectory in red.

Figures S1G-S1J show behavioural data and model fits (M1) of the four participants for which M1 showed the worst fit of the data as measured by the log likelihood.

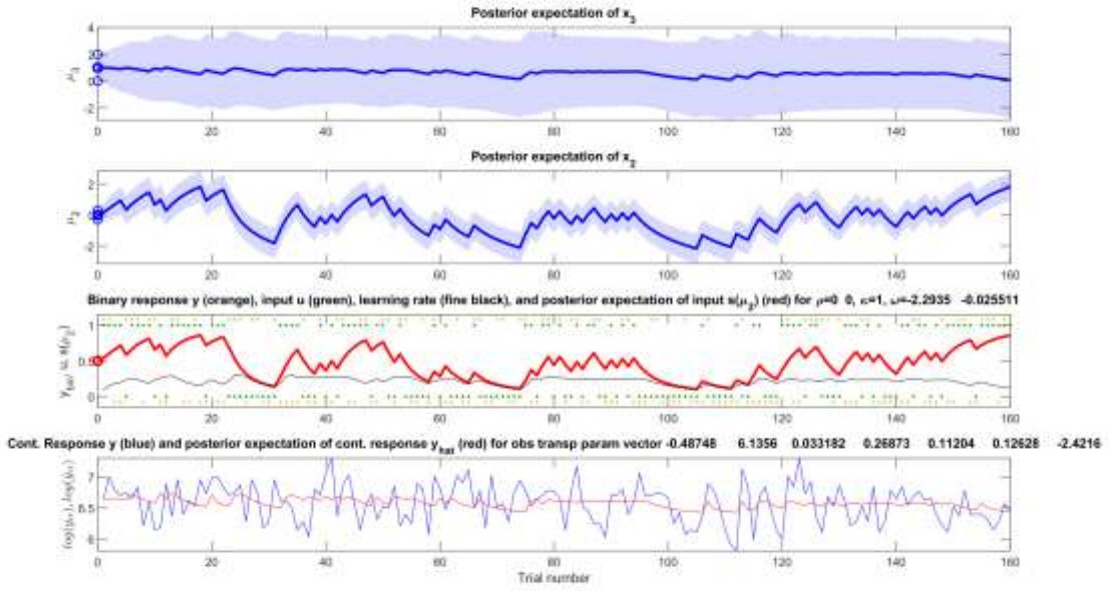

Figure S1G | Behavioural data and model fits (M1) of participant 6 of the main data set. The top panel shows the fitted mean belief μ_3 about the log volatility of the environment in blue with the blue shaded area representing the uncertainty about x_3, i.e., the variance σ_3. The second panel shows mean μ_2 and variance σ_2 of the belief about the cue-outcome contingency. The second lowest panel shows in red the mean belief about the outcome $\hat{\mu}_1$ given one of the two fractals, the actual outcomes for one of the two fractals as green dots (1 = reward, 0 = no reward) and which of the two fractals was selected as yellow dots. The fine black line represents an implied learning rate at the level of the outcome calculated as $\mathbb{1}_{\{\delta_1 \neq 0\}}\left(\frac{\Delta s(\mu_2)}{u - \Delta s(\mu_2)}\right)$ where s is the sigmoid function. The lowest panel shows the empirical log RTs [ms] as blue line and the fitted log RT trajectory in red.

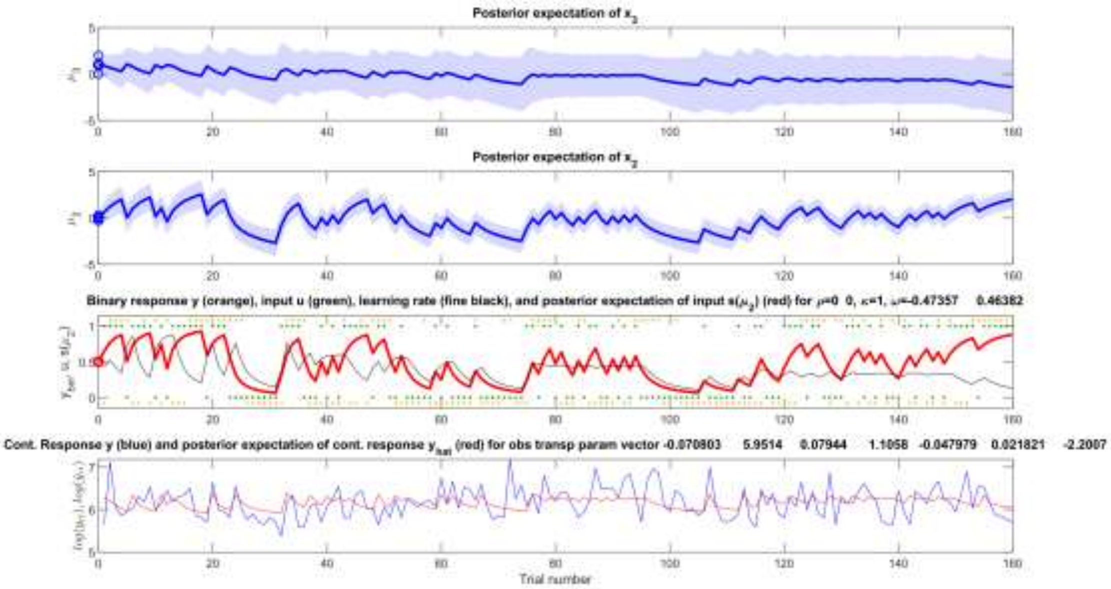

Figure S1H | Behavioural data and model fits (M1) of participant 18 of the main data set. The top panel shows the fitted mean belief μ_3 about the log volatility of the environment in blue with the blue shaded area representing the uncertainty about x_3, i.e., the variance σ_3. The second panel shows mean μ_2 and variance σ_2 of the belief about the cue-outcome contingency. The second lowest panel shows in red the mean belief about the outcome $\hat{\mu}_1$ given one of the two fractals, the actual outcomes for one of the two fractals as green dots (1 = reward, 0 = no reward) and which of the two fractals was selected as yellow dots. The fine black line represents an implied learning rate at the level of the outcome calculated as $\mathbb{1}_{\{\delta_1 \neq 0\}}\left(\frac{\Delta s(\mu_2)}{u-\Delta s(\mu_2)}\right)$ where s is the sigmoid function. The lowest panel shows the empirical log RTs [ms] as blue line and the fitted log RT trajectory in red.

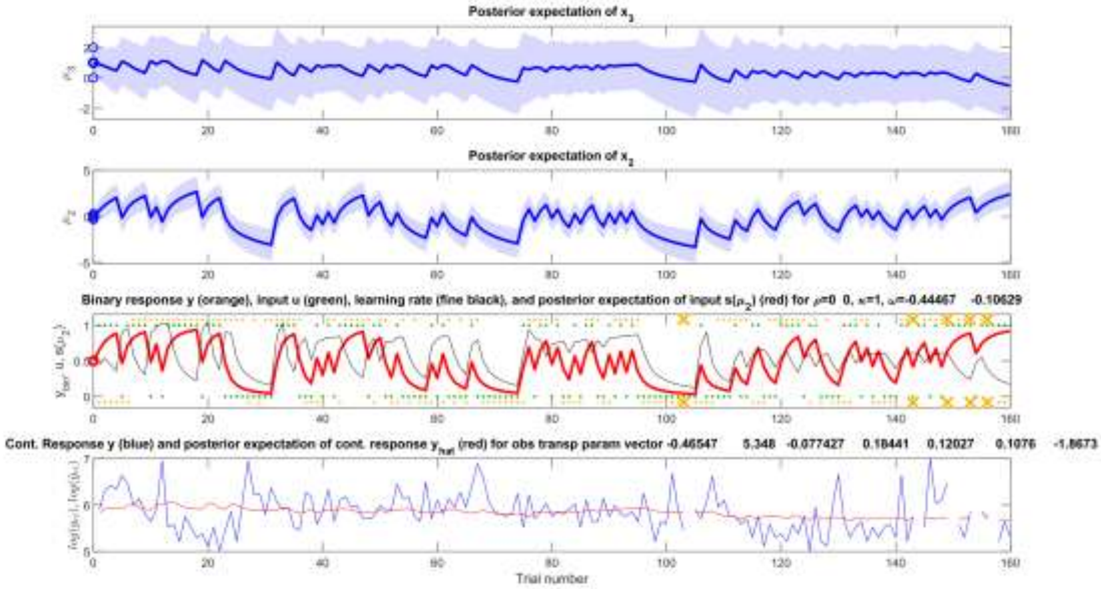

Figure S1I | Behavioural data and model fits (M1) of participant 15 of the main data set. The top panel shows the fitted mean belief μ_3 about the log volatility of the environment in blue with the

blue shaded area representing the uncertainty about x_3, i.e., the variance σ_3. The second panel shows mean μ_2 and variance σ_2 of the belief about the cue-outcome contingency. The second lowest panel shows in red the mean belief about the outcome $\hat{\mu}_1$ given one of the two fractals, the actual outcomes for one of the two fractals as green dots (1 = reward, 0 = no reward) and which of the two fractals was selected as yellow dots. The fine black line represents an implied learning rate at the level of the outcome calculated as $\mathbb{1}_{\{\delta_1 \neq 0\}} \left(\frac{\Delta s(\mu_2)}{u - \Delta s(\mu_2)} \right)$ where s is the sigmoid function. The lowest panel shows the empirical log RTs [ms] as blue line and the fitted log RT trajectory in red.

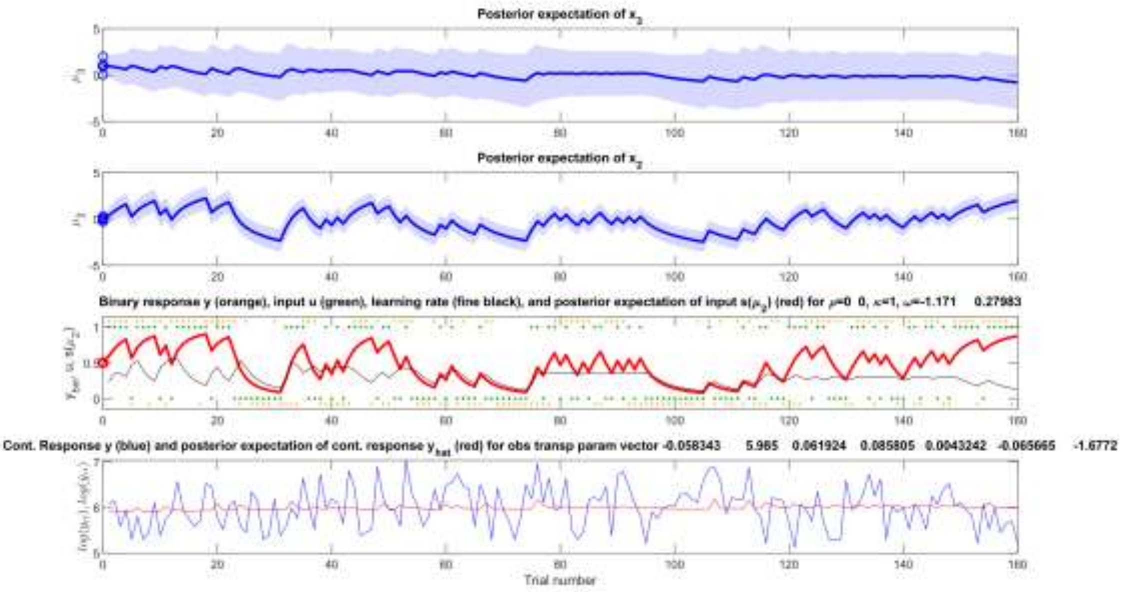

Figure S1J | Behavioural data and model fits (M1) of participant 5 of the main data set. The top panel shows the fitted mean belief μ_3 about the log volatility of the environment in blue with the blue shaded area representing the uncertainty about x_3, i.e., the variance σ_3. The second panel shows mean μ_2 and variance σ_2 of the belief about the cue-outcome contingency. The second lowest panel shows in red the mean belief about the outcome $\hat{\mu}_1$ given one of the two fractals, the actual outcomes for one of the two fractals as green dots (1 = reward, 0 = no reward) and which of the two fractals was selected as yellow dots. The fine black line represents an implied learning rate at the level of the outcome calculated as $\mathbb{1}_{\{\delta_1 \neq 0\}} \left(\frac{\Delta s(\mu_2)}{u - \Delta s(\mu_2)} \right)$ where s is the sigmoid function. The lowest panel shows the empirical log RTs [ms] as blue line and the fitted log RT trajectory in red.

S2. *Empirical priors*

S2a. *Empirical prior densities of M1-M7*

Both *initial* and *empirical prior* densities for all seven models are shown in Figures S2a1-S2a7.

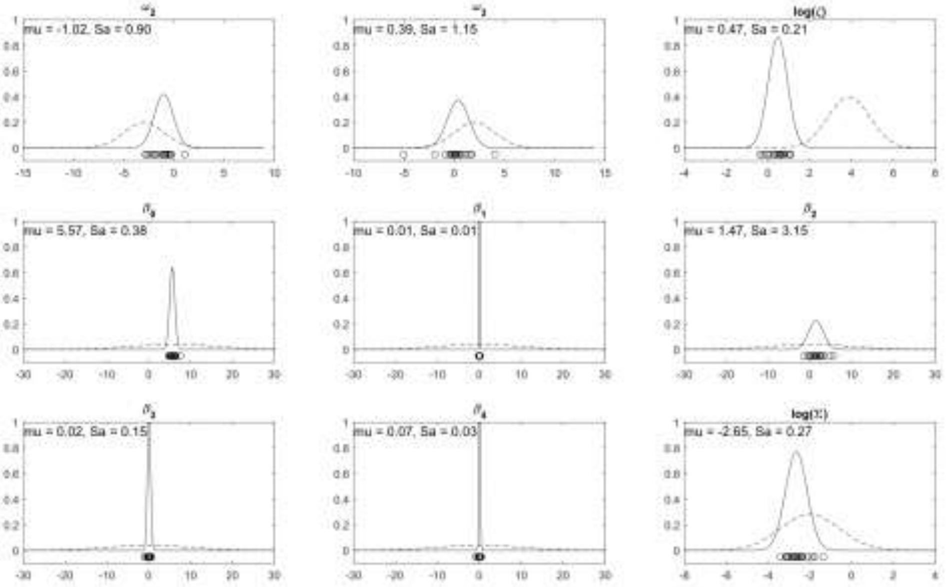

Figure S2a1 | *Empirical prior* densities of M1. For each free parameter of the model, the *initial prior* densities are shown as dashed line and the MAP estimates obtained from model inversion on the held-out data set (N_{pilot}) under the *initial priors* as black circles. The estimated *empirical priors* are shown as black line.

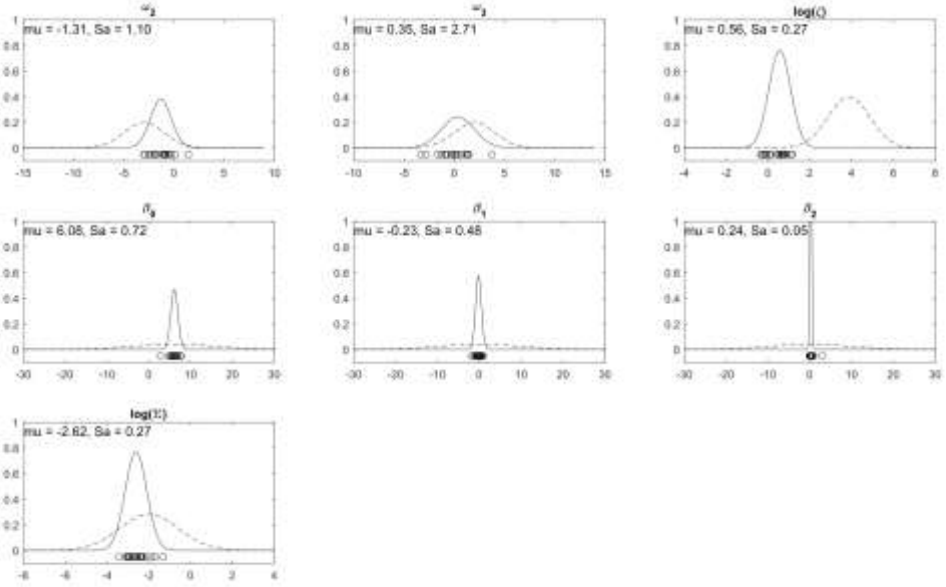

Figure S2a2 | *Empirical prior* densities of M2. For each free parameter of the model, the *initial prior* densities are shown as dashed line and the MAP estimates obtained from model inversion on the held-out data set (N_{pilot}) under the *initial priors* as black circles. The estimated *empirical priors* are shown as black line.

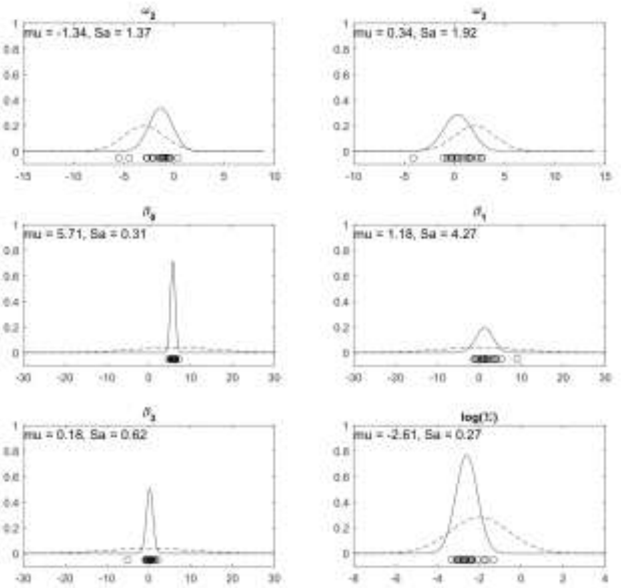

Figure S2a3 | *Empirical prior* densities of M3. For each free parameter of the model, the *initial prior* densities are shown as dashed line and the MAP estimates obtained from model inversion on the held-out data set (N_{pilot}) under the *initial priors* as black circles. The estimated *empirical priors* are shown as black line.

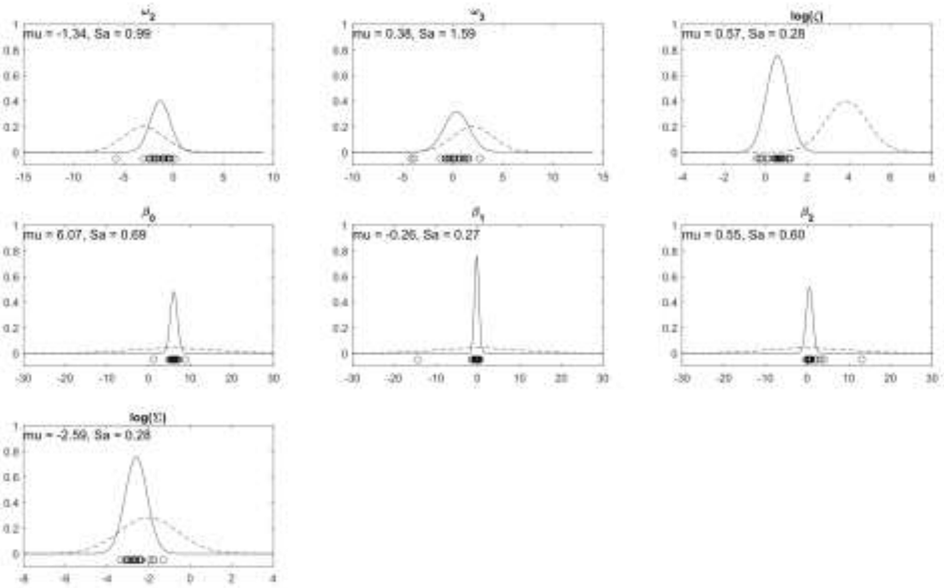

Figure S2a4 | *Empirical prior* densities of M4. For each free parameter of the model, the *initial*

prior densities are shown as dashed line and the MAP estimates obtained from model inversion on the held-out data set (N_{pilot}) under the *initial priors* as black circles. The estimated *empirical priors* are shown as black line.

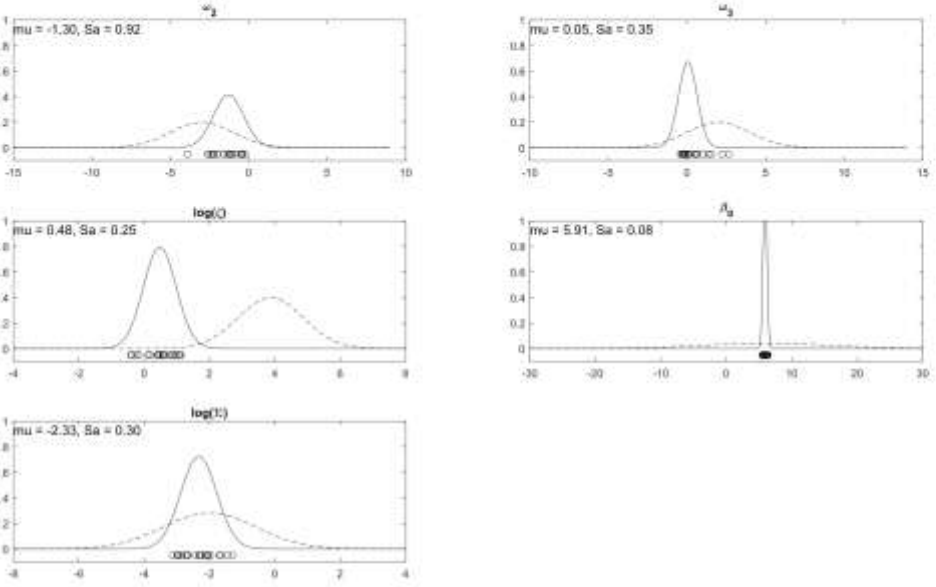

Figure S2a5 | *Empirical prior* densities of M5. For each free parameter of the model, the *initial prior* densities are shown as dashed line and the MAP estimates obtained from model inversion on the held-out data set (N_{pilot}) under the *initial priors* as black circles. The estimated *empirical priors* are shown as black line.

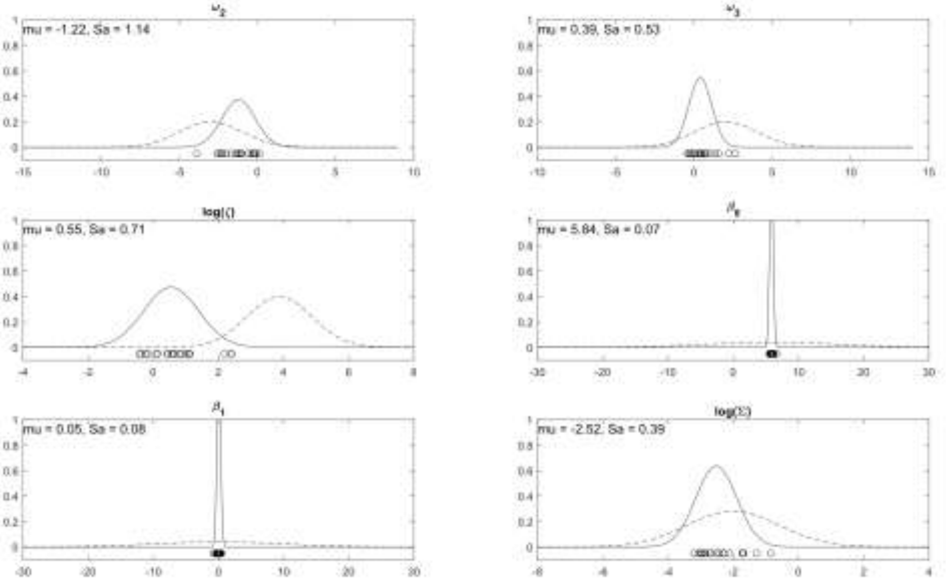

Figure S2a6 | *Empirical prior* densities of M6. For each free parameter of the model, the *initial prior* densities are shown as dashed line and the MAP estimates obtained from model inversion on the held-out data set (N_{pilot}) under the *initial priors* as black circles. The estimated *empirical priors*

are shown as black line.

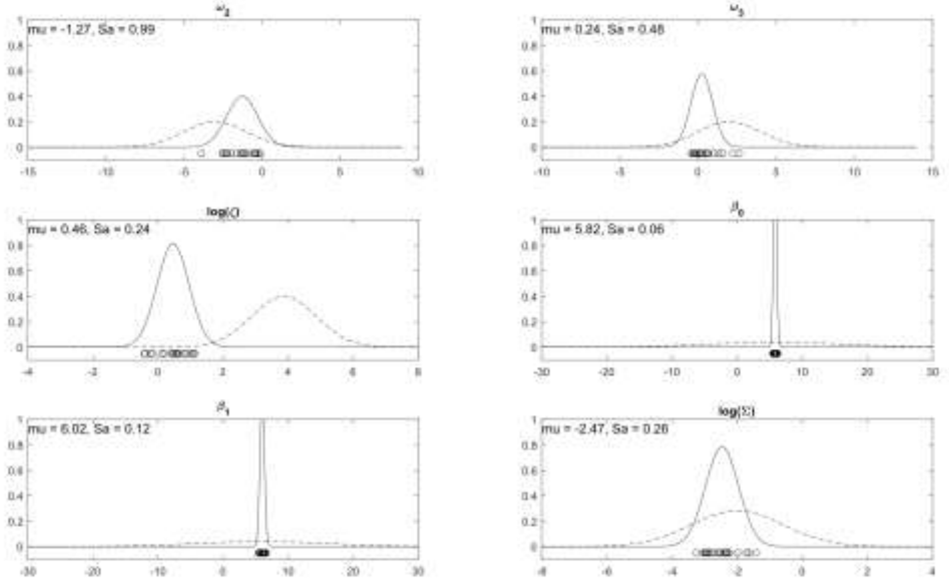

Figure S2a7 | *Empirical prior* densities of M7. For each free parameter of the model, the *initial prior* densities are shown as dashed line and the MAP estimates obtained from model inversion on the held-out data set (N_{pilot}) under the *initial priors* as black circles. The estimated *empirical priors* are shown as black line.

S2b. *Empirical prior predictive distributions of M1-M7*

Empirical prior predictive distributions for the binary response modality and eHGF are shown in Figures S2b1-S2b7.

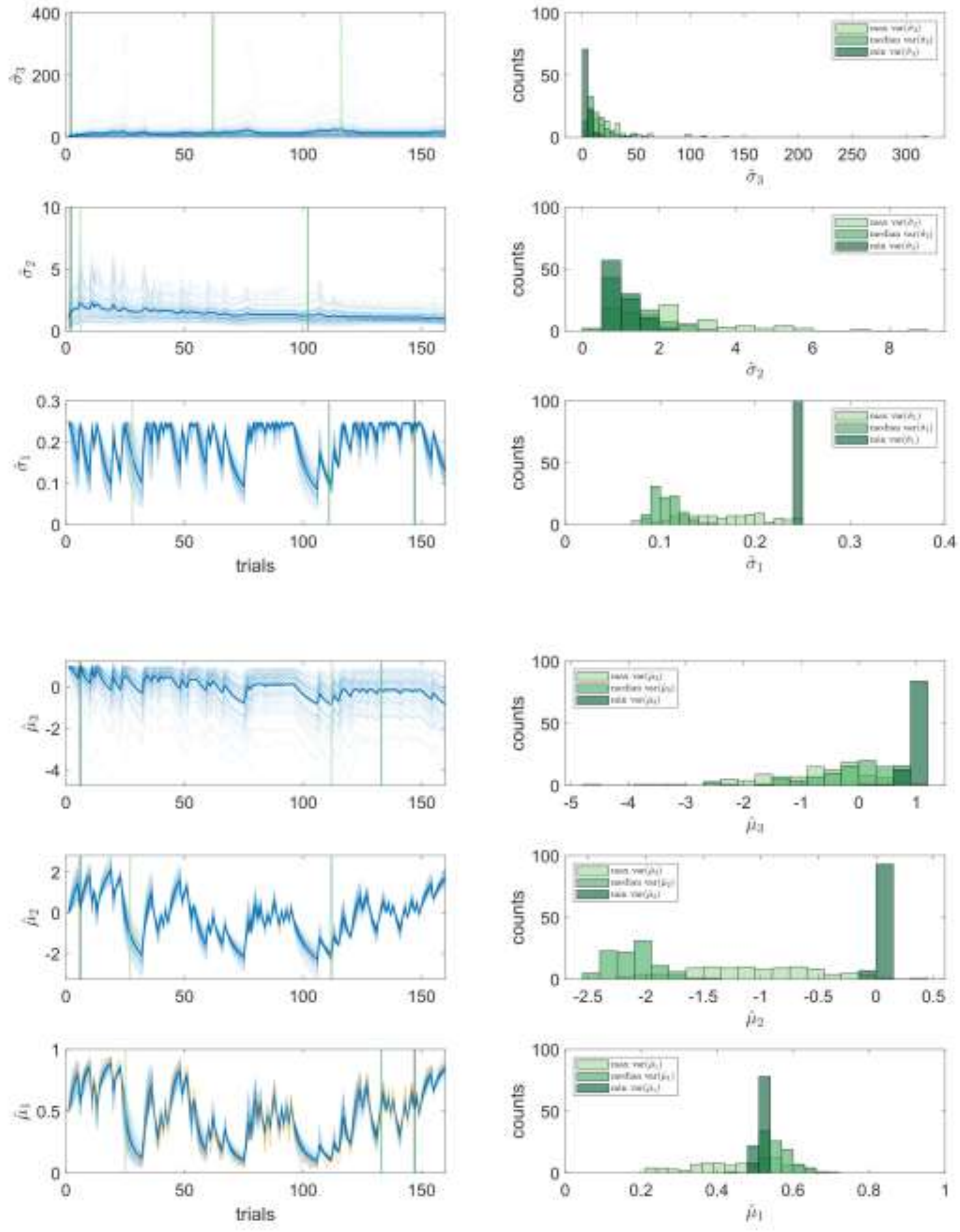

Figure S2b1 | *Empirical prior* predictive distribution of M1 (binary response modality and eHGF). On the left, simulated belief trajectories ($N_{sim} = 100$) are shown for every level of the perceptual model. The mean belief is shown in the lower three left panels whereas the uncertainty (variance) of the belief is shown in the upper three left panels. The thick blue line represents the average over all

simulated belief trajectories at every level. The green vertical lines indicate trials with minimum (dark green), median (green), and maximum (light green) variance across the simulated trajectories as illustrated on the right. In the lower left panel, the yellow line represents the average simulated binary response. On the right, histograms of simulated mean beliefs and uncertainties are presented in green for trials with maximum, median and minimum variance (from light to dark) across simulated trajectories. See the traces on the left for an indication where these trials are located within the trajectory.

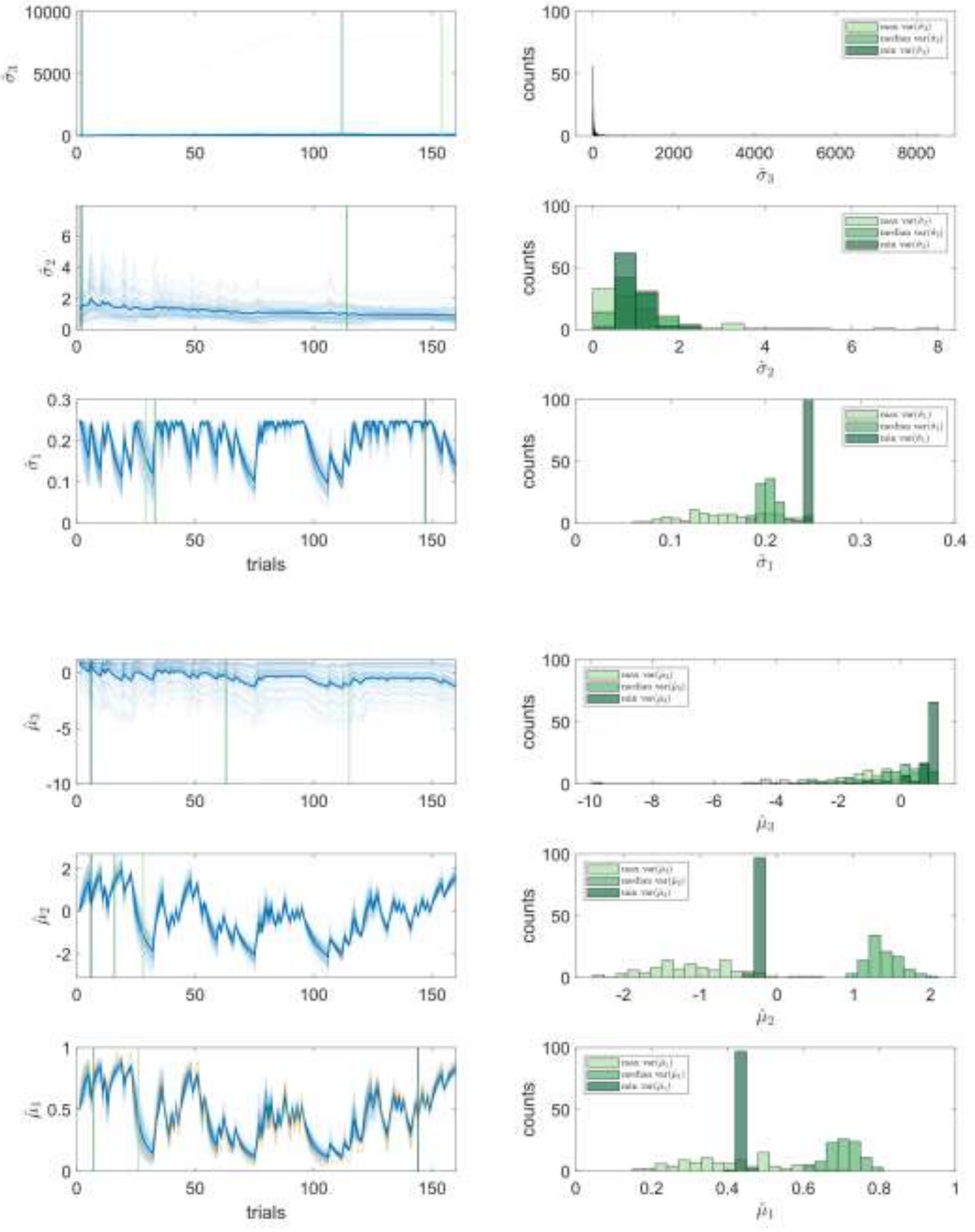

Figure S2b2 | *Empirical prior* predictive distribution of M2 (binary response modality and eHGF). On the left, simulated belief trajectories ($N_{sim} = 100$) are shown for every level of the perceptual model. The mean belief is shown in the lower three left panels whereas the uncertainty (variance) of the belief is shown in the upper three left panels. The thick blue line represents the average over all

simulated belief trajectories at every level. The green vertical lines indicate trials with minimum (dark green), median (green), and maximum (light green) variance across the simulated trajectories as illustrated on the right. In the lower left panel, the yellow line represents the average simulated binary response. On the right, histograms of simulated mean beliefs and uncertainties are presented in green for trials with maximum, median and minimum variance (from light to dark) across simulated trajectories. See the traces on the left for an indication where these trials are located within the trajectory.

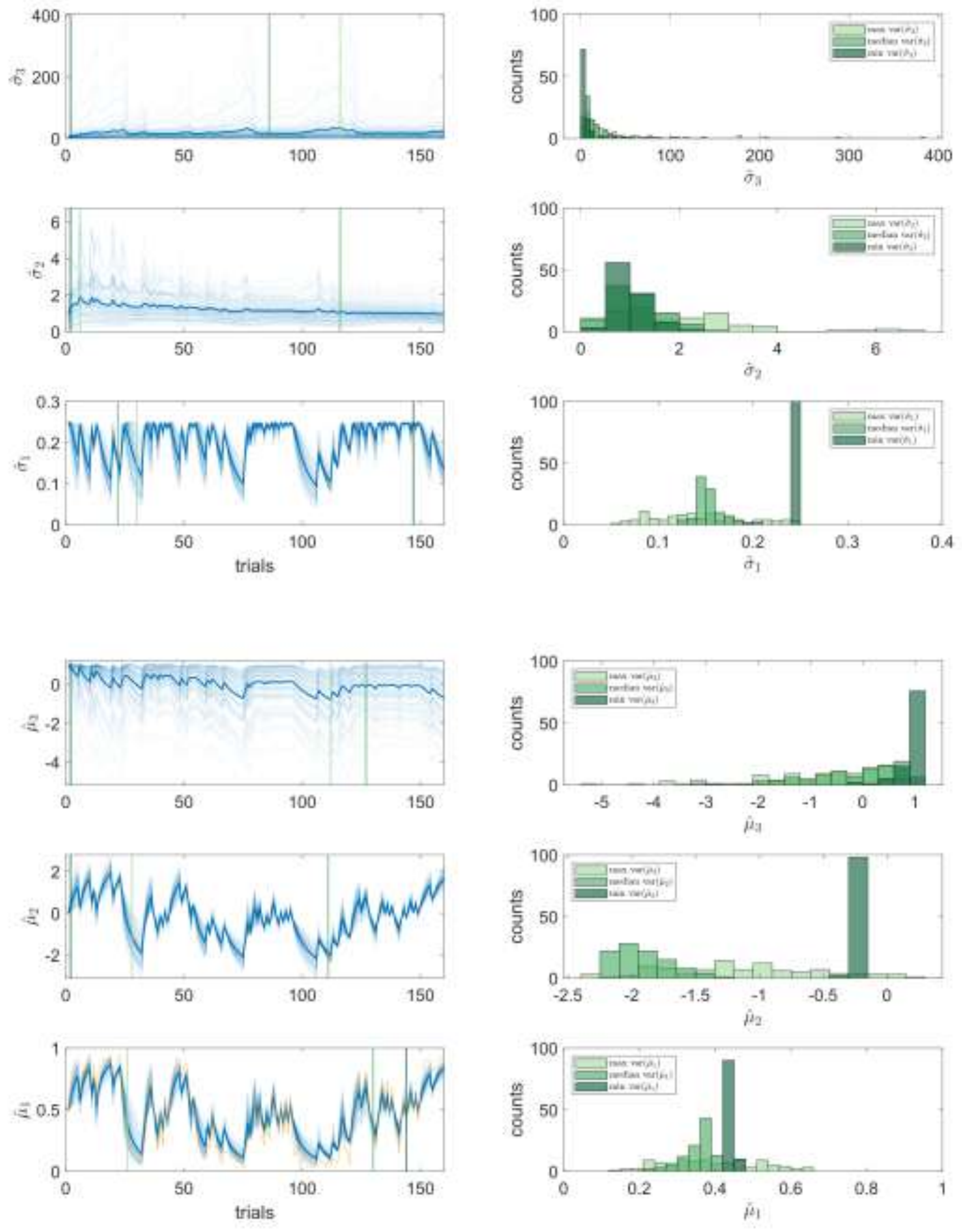

Figure S2b3 | *Empirical prior* predictive distribution of M3 (binary response modality and eHGF). On the left, simulated belief trajectories ($N_{sim} = 100$) are shown for every level of the perceptual model. The mean belief is shown in the lower three left panels whereas the uncertainty (variance) of the belief is shown in the upper three left panels. The thick blue line represents the average over all

simulated belief trajectories at every level. The green vertical lines indicate trials with minimum (dark green), median (green), and maximum (light green) variance across the simulated trajectories as illustrated on the right. In the lower left panel, the yellow line represents the average simulated binary response. On the right, histograms of simulated mean beliefs and uncertainties are presented in green for trials with maximum, median and minimum variance (from light to dark) across simulated trajectories. See the traces on the left for an indication where these trials are located within the trajectory.

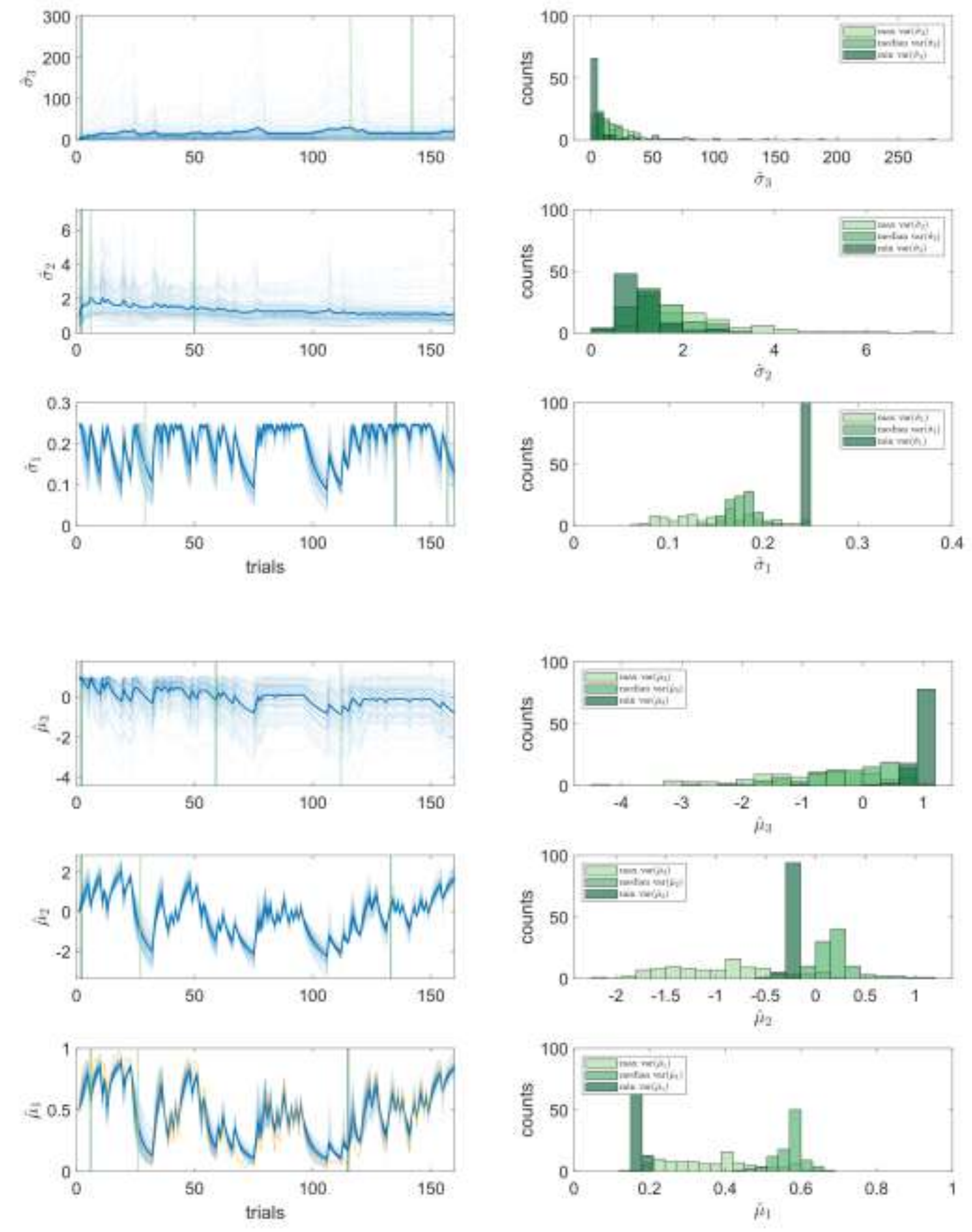

Figure S2b4 | *Empirical prior* predictive distribution of M4 (binary response modality and eHGF). On the left, simulated belief trajectories ($N_{sim} = 100$) are shown for every level of the perceptual model. The mean belief is shown in the lower three left panels whereas the uncertainty (variance) of the belief is shown in the upper three left panels. The thick blue line represents the average over all

simulated belief trajectories at every level. The green vertical lines indicate trials with minimum (dark green), median (green), and maximum (light green) variance across the simulated trajectories as illustrated on the right. In the lower left panel, the yellow line represents the average simulated binary response. On the right, histograms of simulated mean beliefs and uncertainties are presented in green for trials with maximum, median and minimum variance (from light to dark) across simulated trajectories. See the traces on the left for an indication where these trials are located within the trajectory.

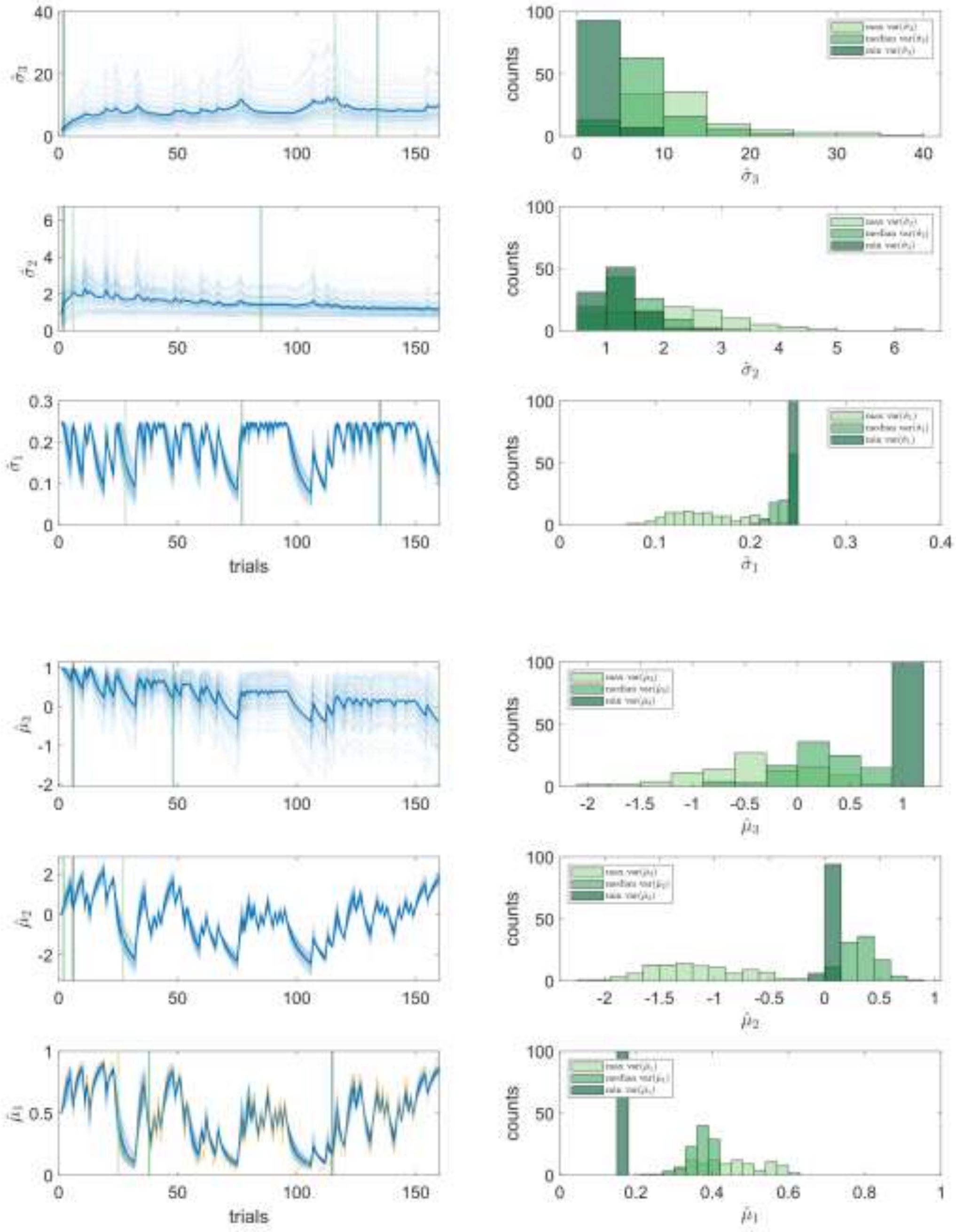

Figure S2b5 | *Empirical prior* predictive distribution of M5 (binary response modality and eHGF). On the left, simulated belief trajectories ($N_{sim} = 100$) are shown for every level of the perceptual model. The mean belief is shown in the lower three left panels whereas the uncertainty (variance) of the belief is shown in the upper three left panels. The thick blue line represents the average over all

simulated belief trajectories at every level. The green vertical lines indicate trials with minimum (dark green), median (green), and maximum (light green) variance across the simulated trajectories as illustrated on the right. In the lower left panel, the yellow line represents the average simulated binary response. On the right, histograms of simulated mean beliefs and uncertainties are presented in green for trials with maximum, median and minimum variance (from light to dark) across simulated trajectories. See the traces on the left for an indication where these trials are located within the trajectory.

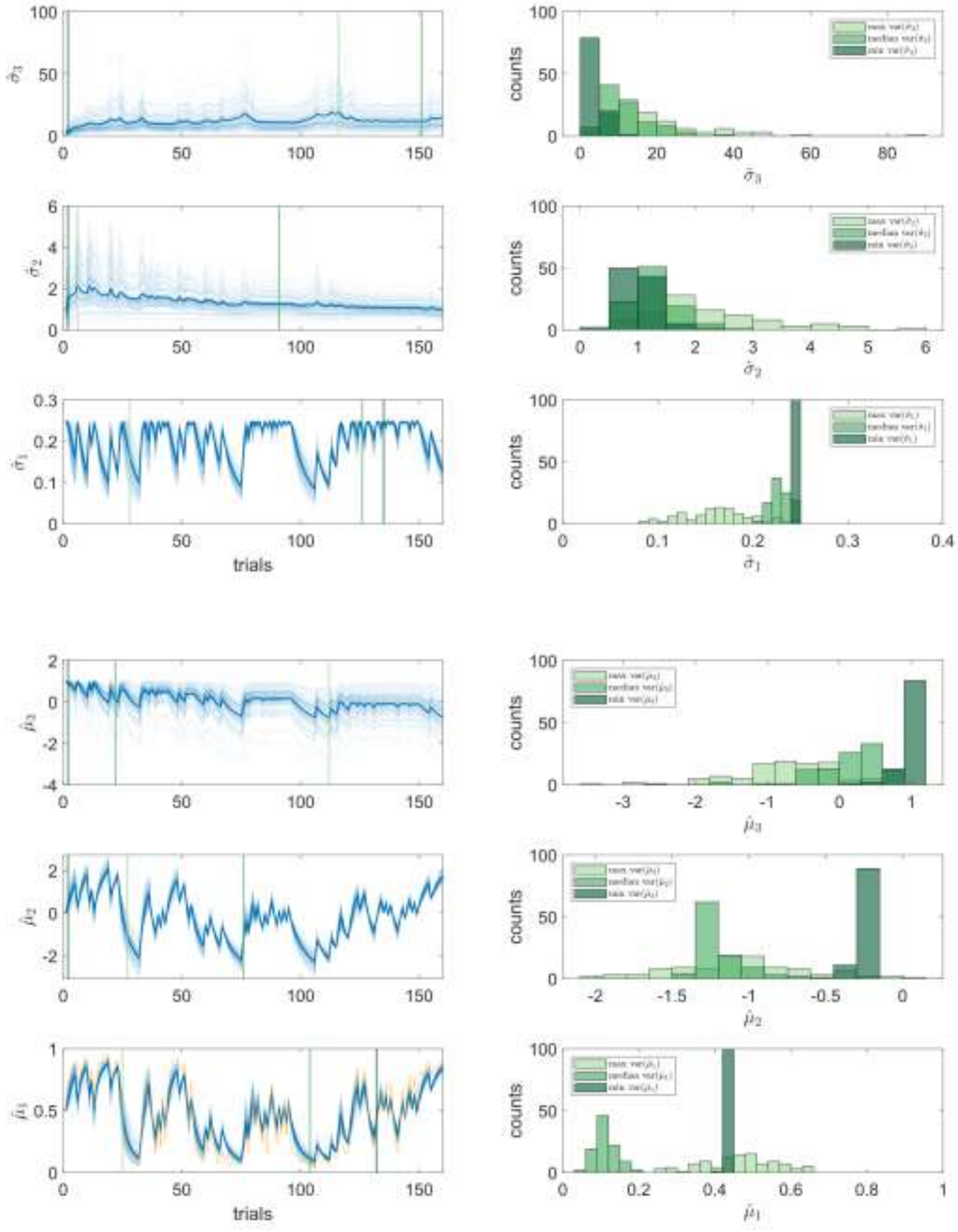

Figure S2b6 | *Empirical prior* predictive distribution of M6 (binary response modality and eHGF). On the left, simulated belief trajectories ($N_{sim} = 100$) are shown for every level of the perceptual model. The mean belief is shown in the lower three left panels whereas the uncertainty (variance) of the belief is shown in the upper three left panels. The thick blue line represents the average over all

simulated belief trajectories at every level. The green vertical lines indicate trials with minimum (dark green), median (green), and maximum (light green) variance across the simulated trajectories as illustrated on the right. In the lower left panel, the yellow line represents the average simulated binary response. On the right, histograms of simulated mean beliefs and uncertainties are presented in green for trials with maximum, median and minimum variance (from light to dark) across simulated trajectories. See the traces on the left for an indication where these trials are located within the trajectory.

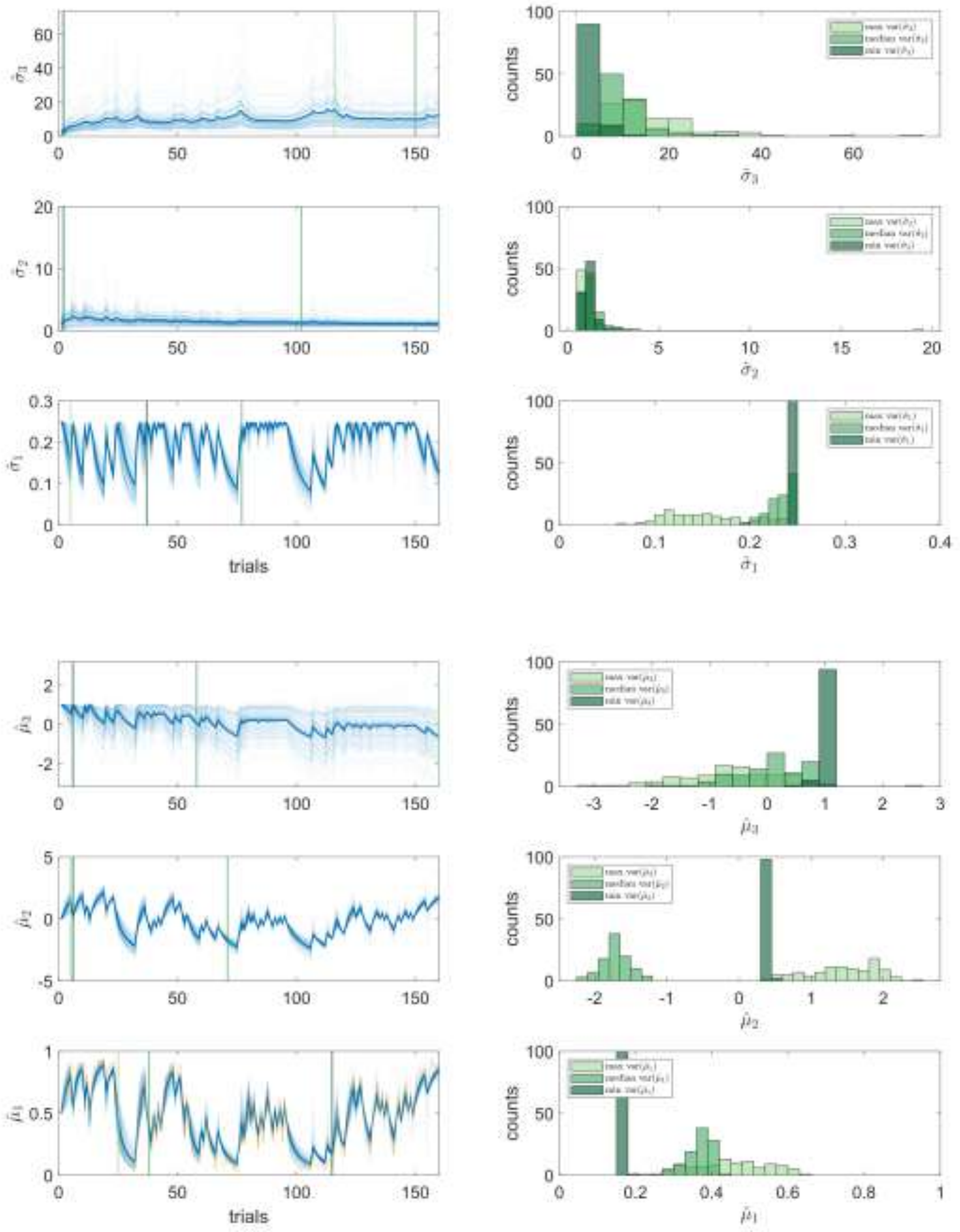

Figure S2b7 | *Empirical prior* predictive distribution of M7 (binary response modality and eHGF). On the left, simulated belief trajectories ($N_{sim} = 100$) are shown for every level of the perceptual model. The mean belief is shown in the lower three left panels whereas the uncertainty (variance) of the belief is shown in the upper three left panels. The thick blue line represents the average over all

simulated belief trajectories at every level. The green vertical lines indicate trials with minimum (dark green), median (green), and maximum (light green) variance across the simulated trajectories as illustrated on the right. In the lower left panel, the yellow line represents the average simulated binary response. On the right, histograms of simulated mean beliefs and uncertainties are presented in green for trials with maximum, median and minimum variance (from light to dark) across simulated trajectories. See the traces on the left for an indication where these trials are located within the trajectory.

Empirical prior predictive distributions for the continuous response modality are shown in Figures S2b8 and S2b9.

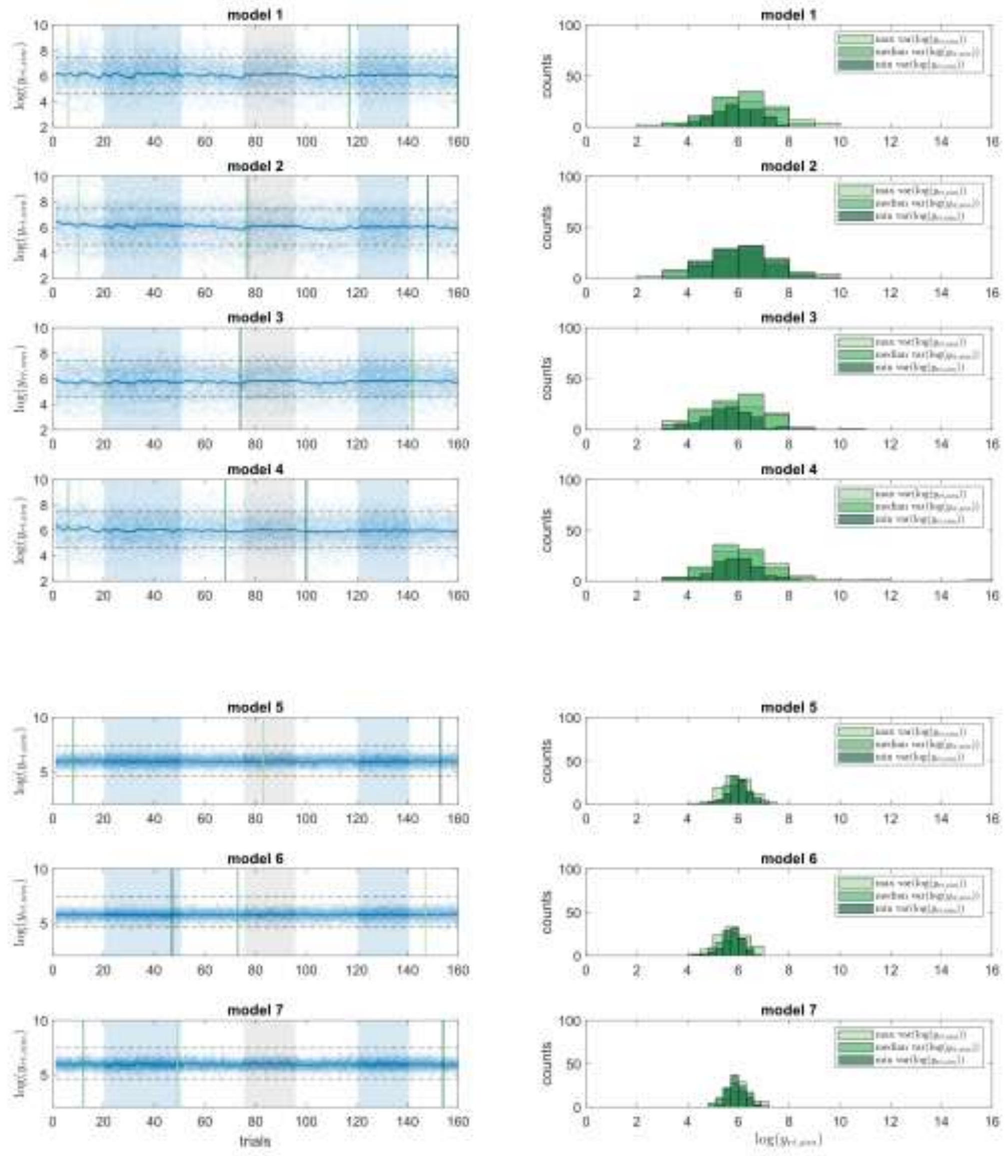

Figure S2b8 | *Empirical prior* predictive distributions of M1-M7 (simulated log RTs). On the left, simulated log RT trajectories are shown in blue with the thick blue line representing the average over all simulated trajectories for each model ($N_{sim} = 100$). The dashed lines represent the boundaries of the response window in the SPIRL task. The green vertical lines indicate trials with minimum (dark green), median (green), and maximum (light green) variance across the simulated trajectories as illustrated on the right. On the right, histograms of simulated log RT data are presented in green for trials with maximum, median and minimum variance (from light to dark) across simulated trajectories. See the traces on the left for an indication where these trials are located within the trajectory.

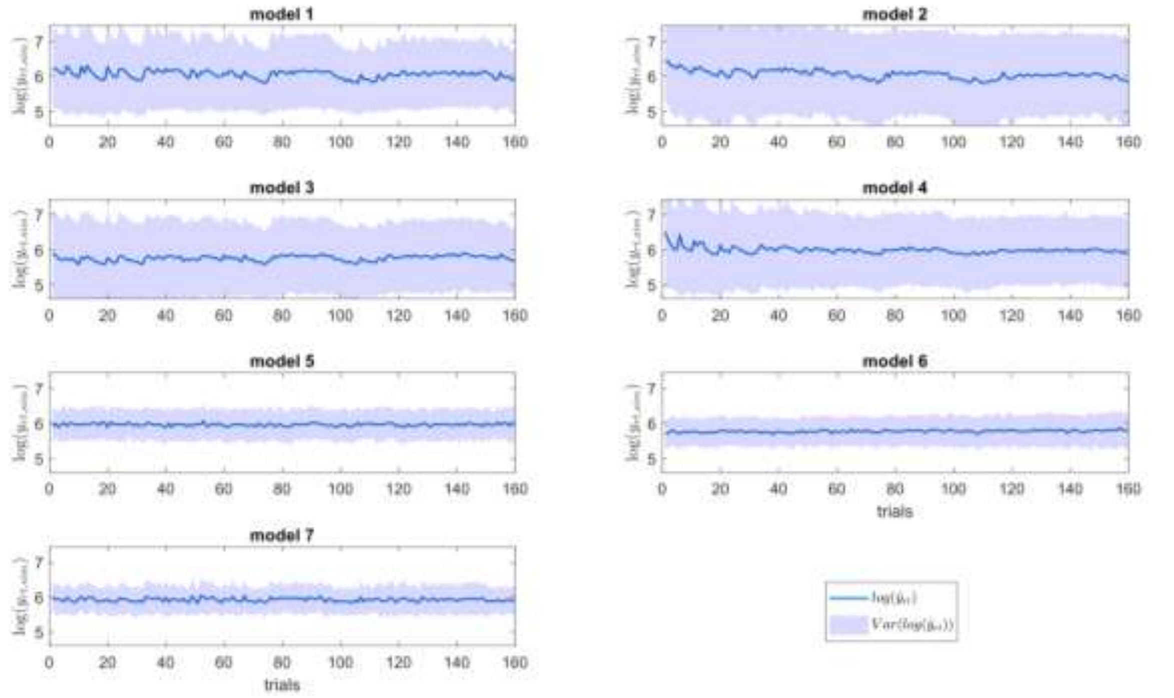

Figure S2b9 | *Empirical prior* predictive distributions of M1-M7 (simulated log RTs). Mean and standard deviation of simulated log RT trajectories for each model are shown in blue ($N_{sim} = 100$).

S2c. *Initial prior predictive distributions of M1-M7*

Initial prior predictive distributions for the binary response modality and eHGF are shown in Figures S2c1-S2c7.

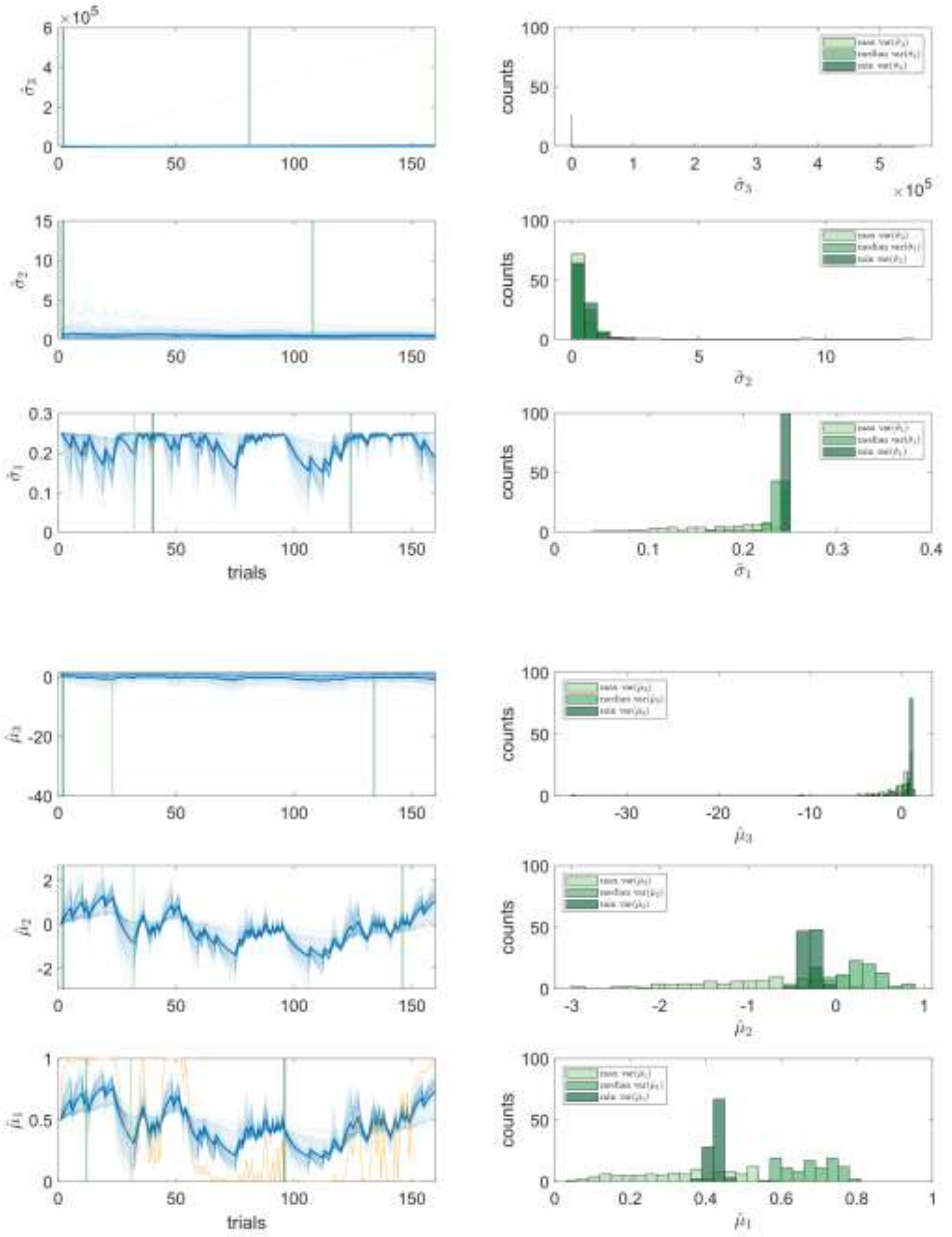

Figure S2c1 | *Initial prior* predictive distribution of M1 (binary response modality and eHGF). On the left, simulated belief trajectories ($N_{sim} = 100$) are shown for every level of the perceptual model. The mean belief is shown in the lower three left panels whereas the uncertainty (variance) of the belief is shown in the upper three left panels. The thick blue line represents the average over all simulated belief trajectories at every level. The green vertical lines indicate trials with minimum (dark green), median (green), and maximum (light green) variance across the simulated trajectories as

illustrated on the right. In the lower left panel, the yellow line represents the average simulated binary response. On the right, histograms of simulated mean beliefs and uncertainties are presented in green for trials with maximum, median and minimum variance (from light to dark) across simulated trajectories. See the traces on the left for an indication where these trials are located within the trajectory.

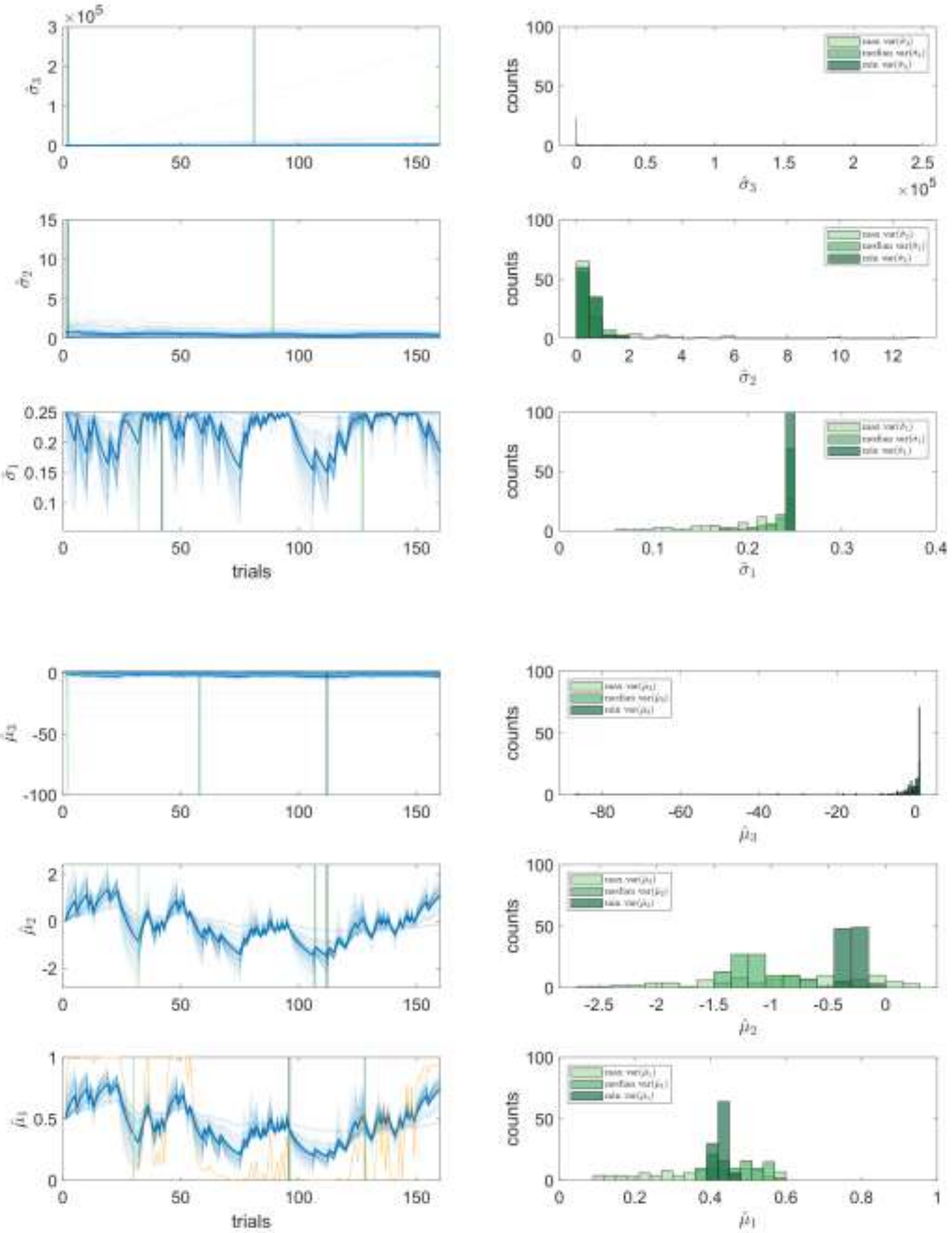

Figure S2c2 | *Initial prior* predictive distribution of M2 (binary response modality and eHGF). On the left, simulated belief trajectories ($N_{sim} = 100$) are shown for every level of the perceptual model. The mean belief is shown in the lower three left panels whereas the uncertainty (variance) of the belief is shown in the upper three left panels. The thick blue line represents the average over all simulated belief trajectories at every level. The green vertical lines indicate trials with minimum (dark

green), median (green), and maximum (light green) variance across the simulated trajectories as illustrated on the right. In the lower left panel, the yellow line represents the average simulated binary response. On the right, histograms of simulated mean beliefs and uncertainties are presented in green for trials with maximum, median and minimum variance (from light to dark) across simulated trajectories. See the traces on the left for an indication where these trials are located within the trajectory.

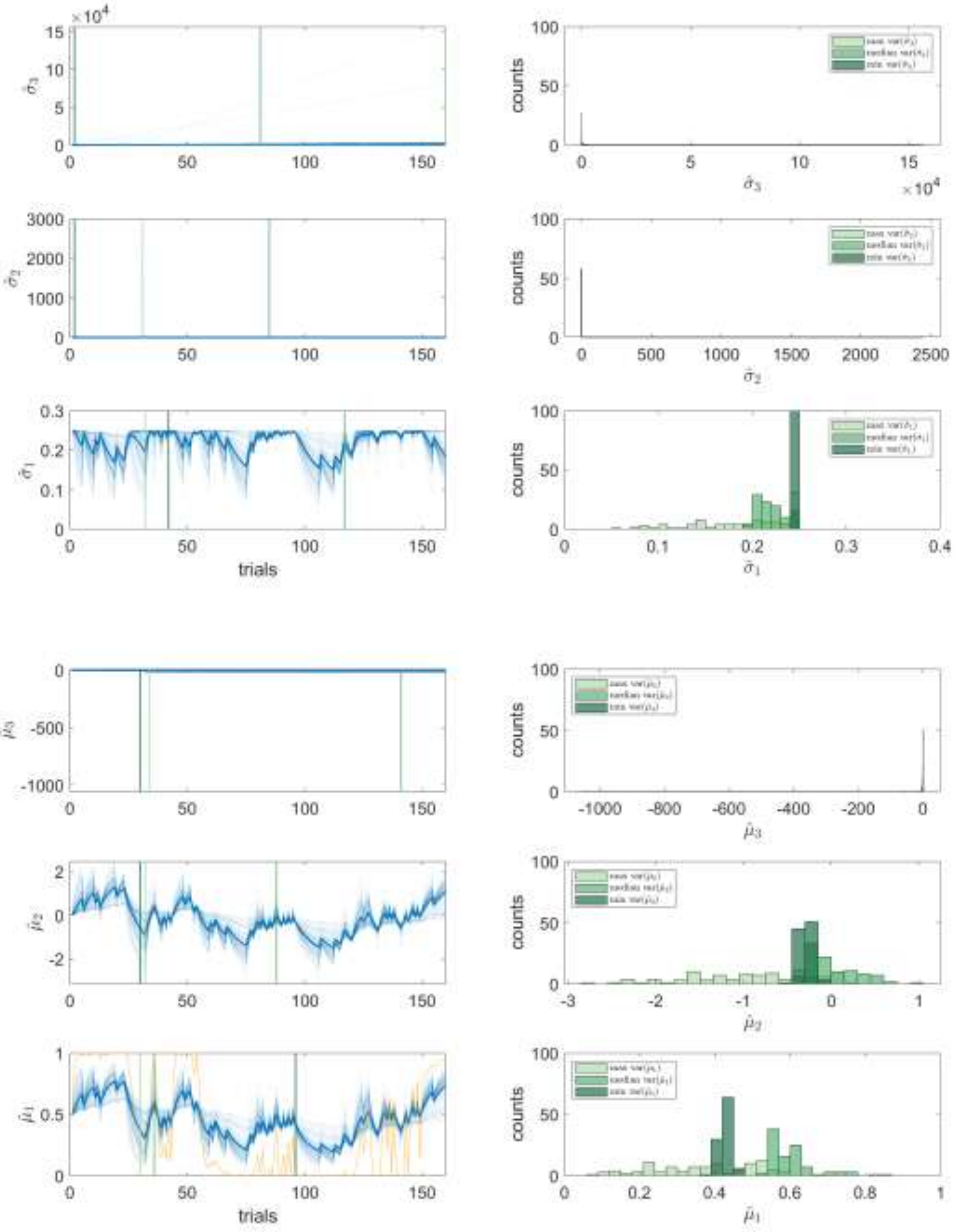

Figure S2c3 | *Initial prior* predictive distribution of M3 (binary response modality and eHGF). On the left, simulated belief trajectories ($N_{sim} = 100$) are shown for every level of the perceptual model. The mean belief is shown in the lower three left panels whereas the uncertainty (variance) of the belief is shown in the upper three left panels. The thick blue line represents the average over all

simulated belief trajectories at every level. The green vertical lines indicate trials with minimum (dark green), median (green), and maximum (light green) variance across the simulated trajectories as illustrated on the right. In the lower left panel, the yellow line represents the average simulated binary response. On the right, histograms of simulated mean beliefs and uncertainties are presented in green for trials with maximum, median and minimum variance (from light to dark) across simulated trajectories. See the traces on the left for an indication where these trials are located within the trajectory.

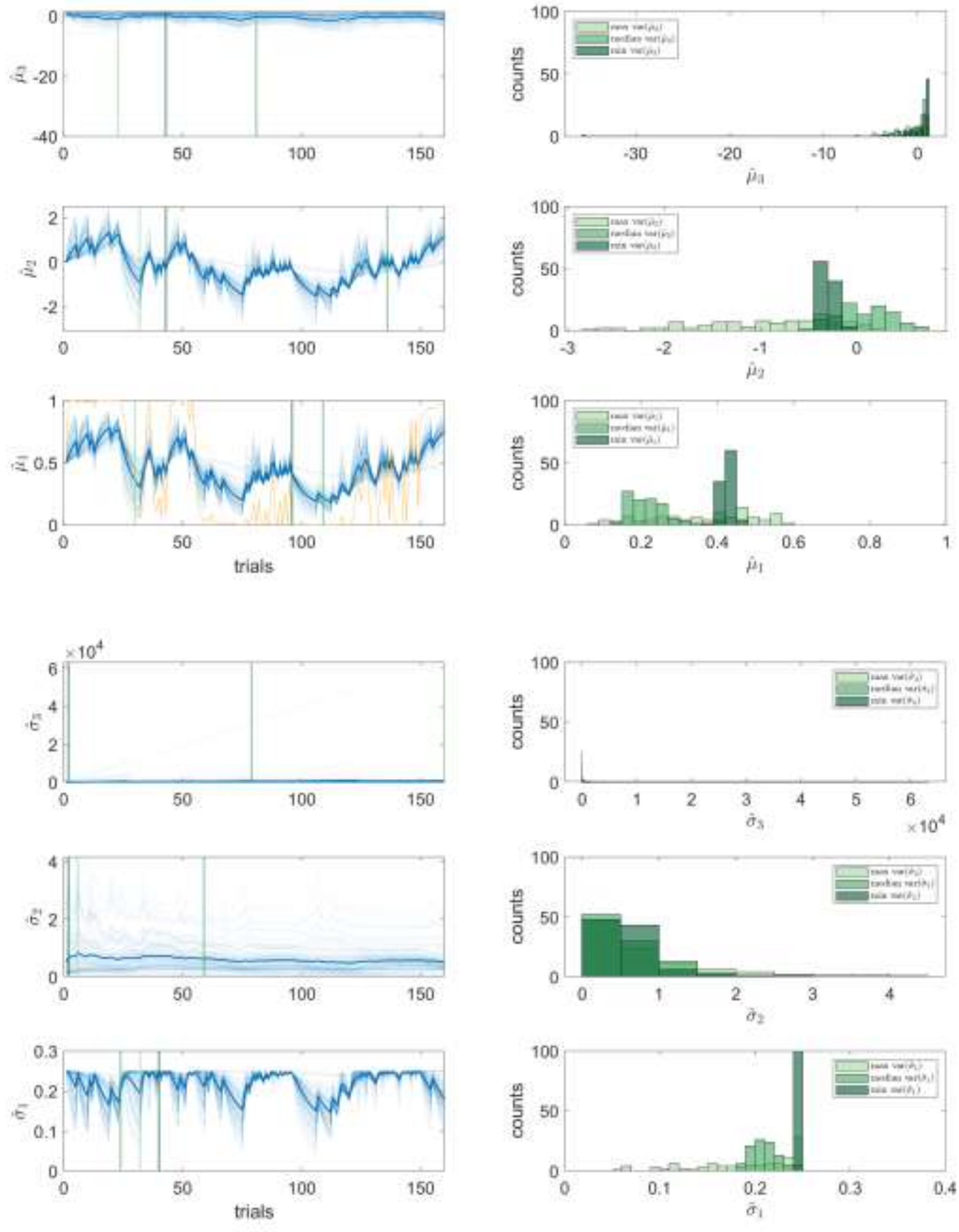

Figure S2c4 | *Initial prior* predictive distribution of M4 (binary response modality and eHGF). On the left, simulated belief trajectories ($N_{sim} = 100$) are shown for every level of the perceptual model. The mean belief is shown in the lower three left panels whereas the uncertainty (variance) of the belief is shown in the upper three left panels. The thick blue line represents the average over all

simulated belief trajectories at every level. The green vertical lines indicate trials with minimum (dark green), median (green), and maximum (light green) variance across the simulated trajectories as illustrated on the right. In the lower left panel, the yellow line represents the average simulated binary response. On the right, histograms of simulated mean beliefs and uncertainties are presented in green for trials with maximum, median and minimum variance (from light to dark) across simulated trajectories. See the traces on the left for an indication where these trials are located within the trajectory.

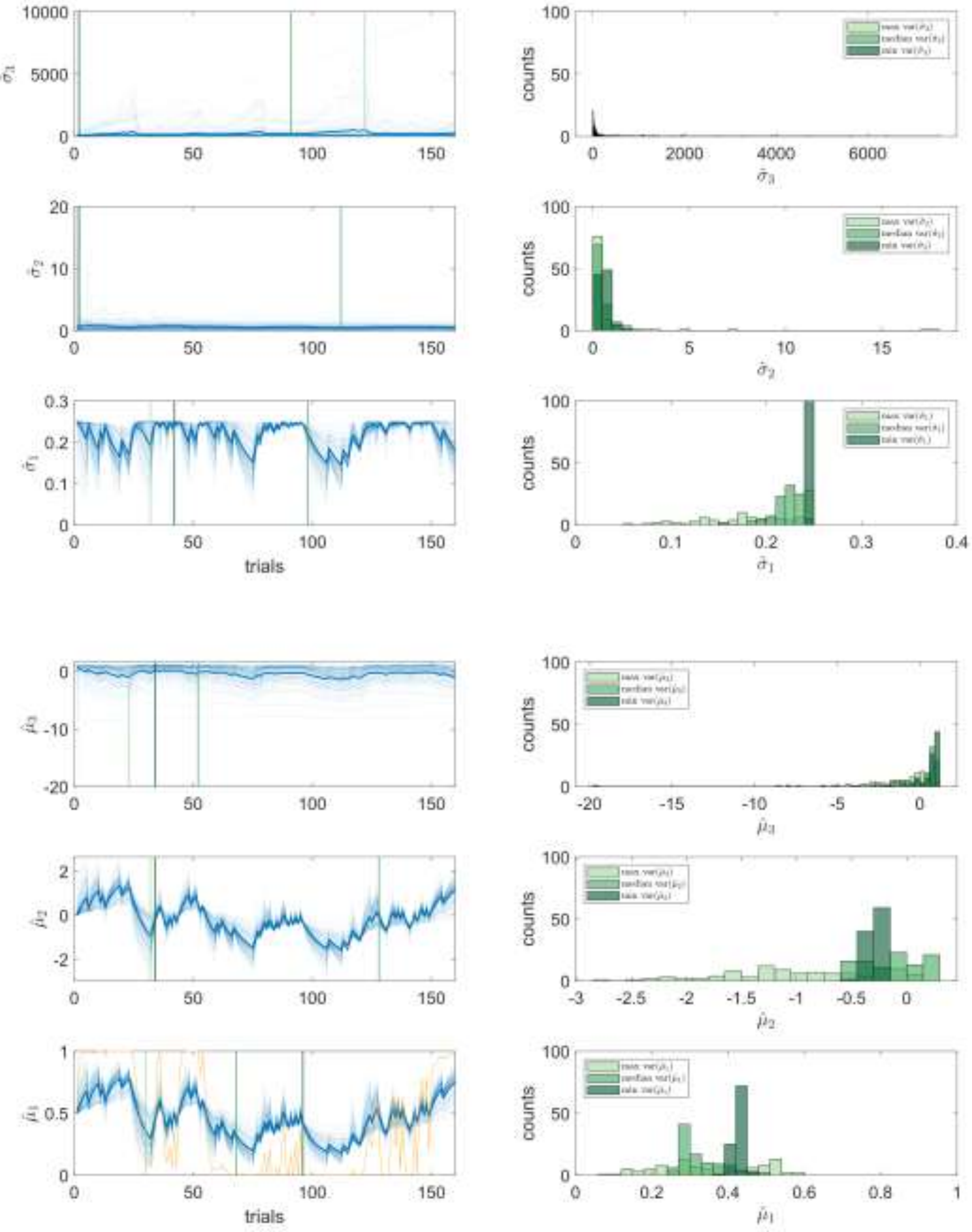

Figure S2c5 | *Initial prior* predictive distribution of M5 (binary response modality and eHGF). On the left, simulated belief trajectories ($N_{sim} = 100$) are shown for every level of the perceptual model. The mean belief is shown in the lower three left panels whereas the uncertainty (variance) of the belief is shown in the upper three left panels. The thick blue line represents the average over all

simulated belief trajectories at every level. The green vertical lines indicate trials with minimum (dark green), median (green), and maximum (light green) variance across the simulated trajectories as illustrated on the right. In the lower left panel, the yellow line represents the average simulated binary response. On the right, histograms of simulated mean beliefs and uncertainties are presented in green for trials with maximum, median and minimum variance (from light to dark) across simulated trajectories. See the traces on the left for an indication where these trials are located within the trajectory.

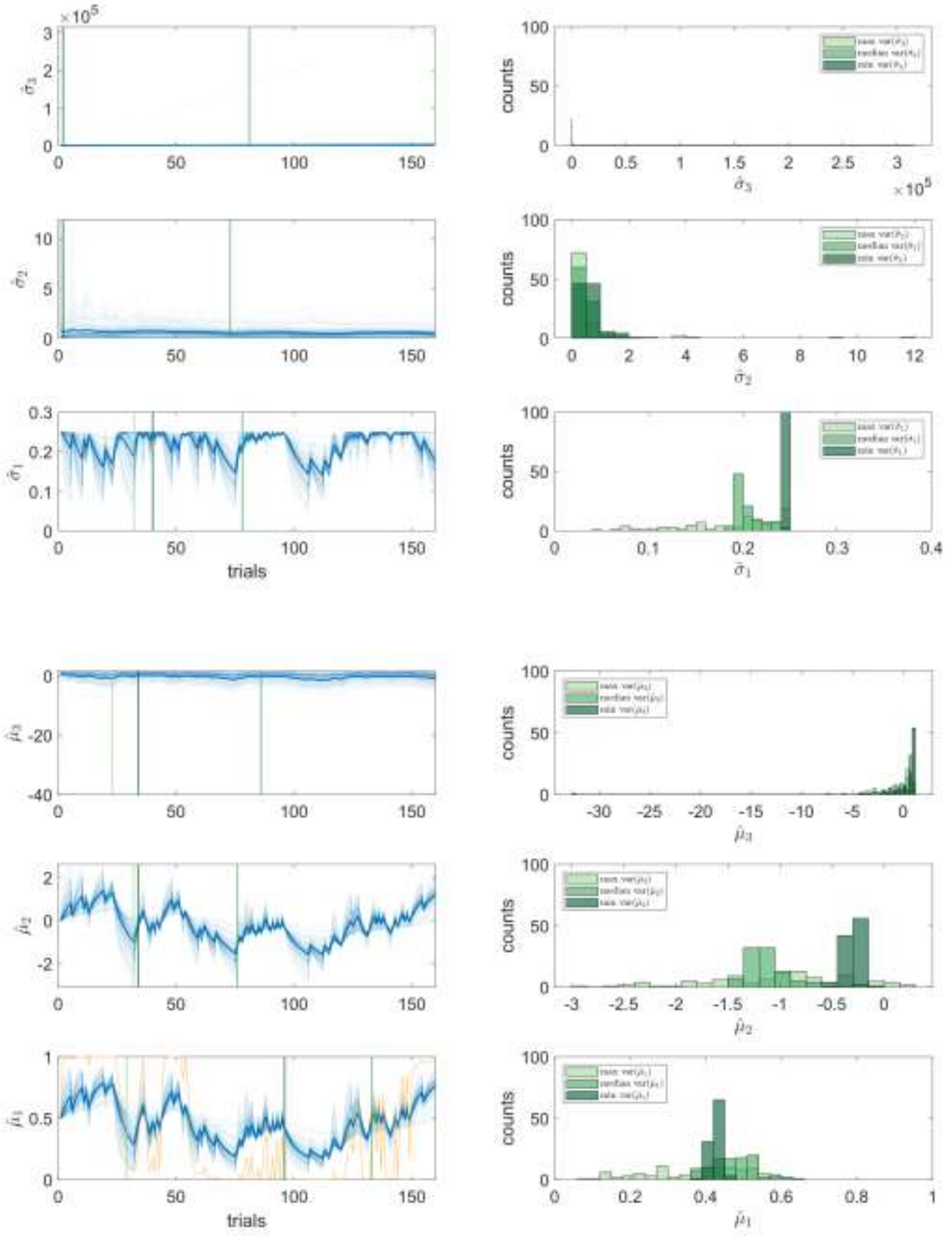

Figure S2c6 | *Initial prior* predictive distribution of M6 (binary response modality and eHGF). On the left, simulated belief trajectories ($N_{sim} = 100$) are shown for every level of the perceptual model. The mean belief is shown in the lower three left panels whereas the uncertainty (variance) of the belief is shown in the upper three left panels. The thick blue line represents the average over all

simulated belief trajectories at every level. The green vertical lines indicate trials with minimum (dark green), median (green), and maximum (light green) variance across the simulated trajectories as illustrated on the right. In the lower left panel, the yellow line represents the average simulated binary response. On the right, histograms of simulated mean beliefs and uncertainties are presented in green for trials with maximum, median and minimum variance (from light to dark) across simulated trajectories. See the traces on the left for an indication where these trials are located within the trajectory.

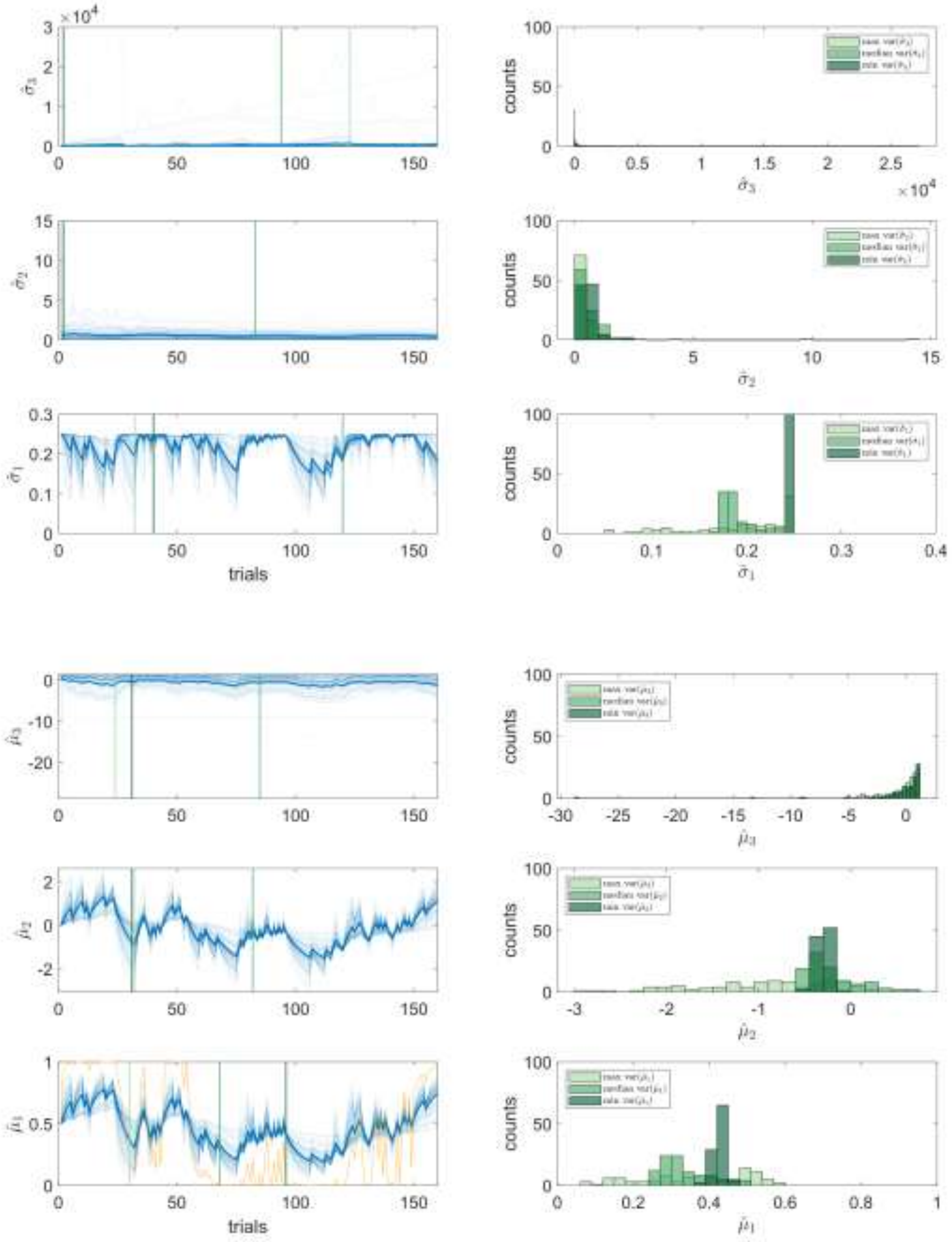

Figure S2c7 | *Initial prior* predictive distribution of M7 (binary response modality and eHGF). On the left, simulated belief trajectories ($N_{sim} = 100$) are shown for every level of the perceptual model. The mean belief is shown in the lower three left panels whereas the uncertainty (variance) of the belief is shown in the upper three left panels. The thick blue line represents the average over all

simulated belief trajectories at every level. The green vertical lines indicate trials with minimum (dark green), median (green), and maximum (light green) variance across the simulated trajectories as illustrated on the right. In the lower left panel, the yellow line represents the average simulated binary response. On the right, histograms of simulated mean beliefs and uncertainties are presented in green for trials with maximum, median and minimum variance (from light to dark) across simulated trajectories. See the traces on the left for an indication where these trials are located within the trajectory.

Initial prior predictive distributions for the continuous response modality are shown in Figure S2c8.

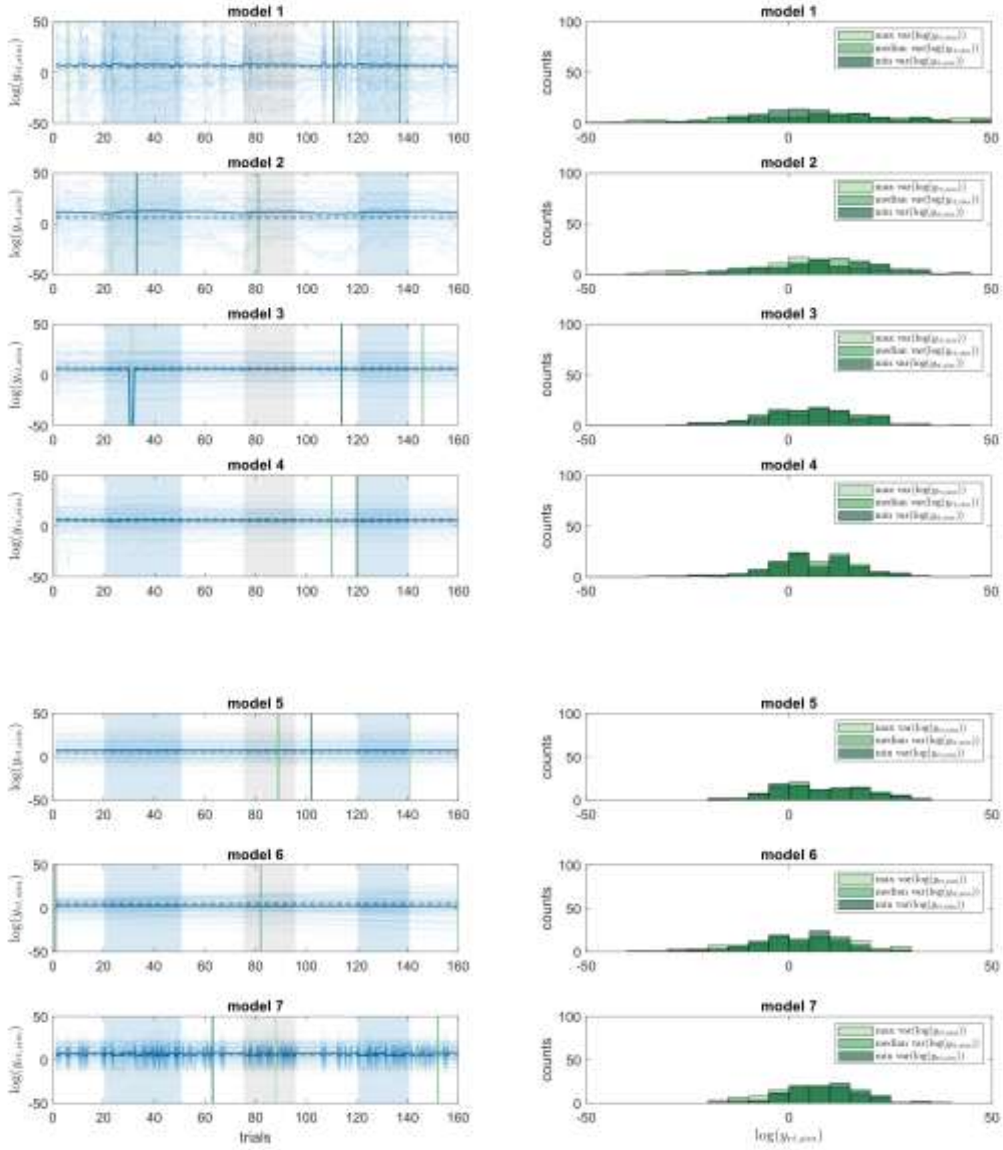

Figure S2c8 | *Initial prior* predictive distributions of M1-M7 (simulated log RTs). On the left, simulated log RT trajectories are shown in blue with the thick blue line representing the average over all simulated trajectories for each model ($N_{sim} = 100$). The dashed lines represent the boundaries of the response window in the SPIRL task. The green vertical lines indicate trials with minimum (dark green), median (green), and maximum (light green) variance across the simulated trajectories as illustrated on the right. On the right, histograms of simulated log RT data are presented in green for trials with maximum, median and minimum variance (from light to dark) across simulated trajectories. See the traces on the left for an indication where these trials are located within the trajectory.

S3: Parameter recovery results of M2-M7

Results from the parameter recovery analysis of M2-M7 are shown in Figures S3A-S3F.

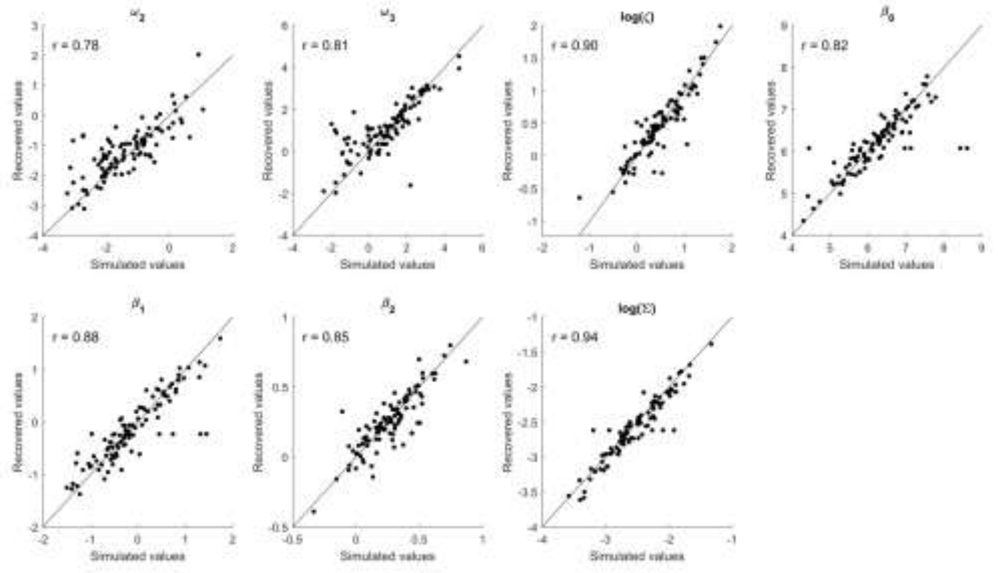

Figure S3A | Parameter recovery of M2. $N_{sim} = 100$ parameter values are displayed (simulated parameter values on the x-axis, fitted parameter values on the y-axis) and Pearson correlation coefficients between simulated and estimated parameter values for each free parameter are denoted by r. The black line is the identity line representing perfect recovery.

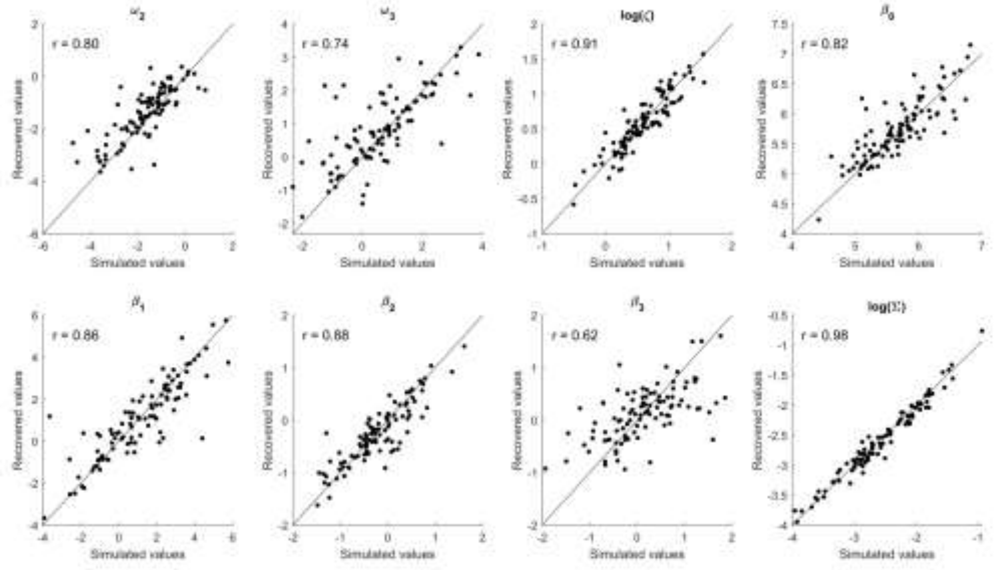

Figure S3B | Parameter recovery of M3. $N_{sim} = 100$ parameter values are displayed (simulated parameter values on the x-axis, fitted parameter values on the y-axis) and Pearson correlation coefficients between simulated and estimated parameter values for each free parameter

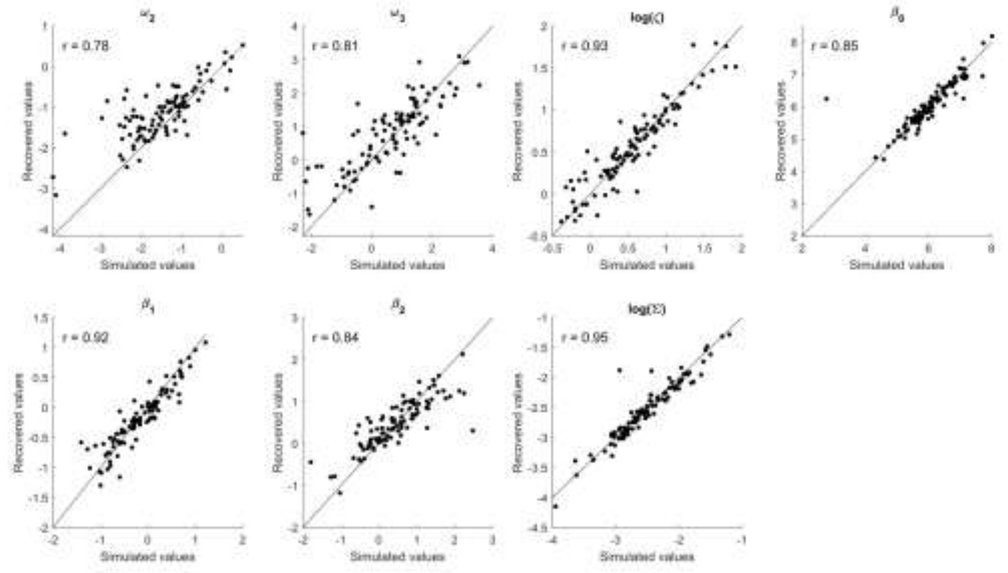

Figure S3C | Parameter recovery of M4. $N_{sim} = 100$ parameter values are displayed (simulated parameter values on the x-axis, fitted parameter values on the y-axis) and Pearson correlation coefficients between simulated and estimated parameter values for each free parameter

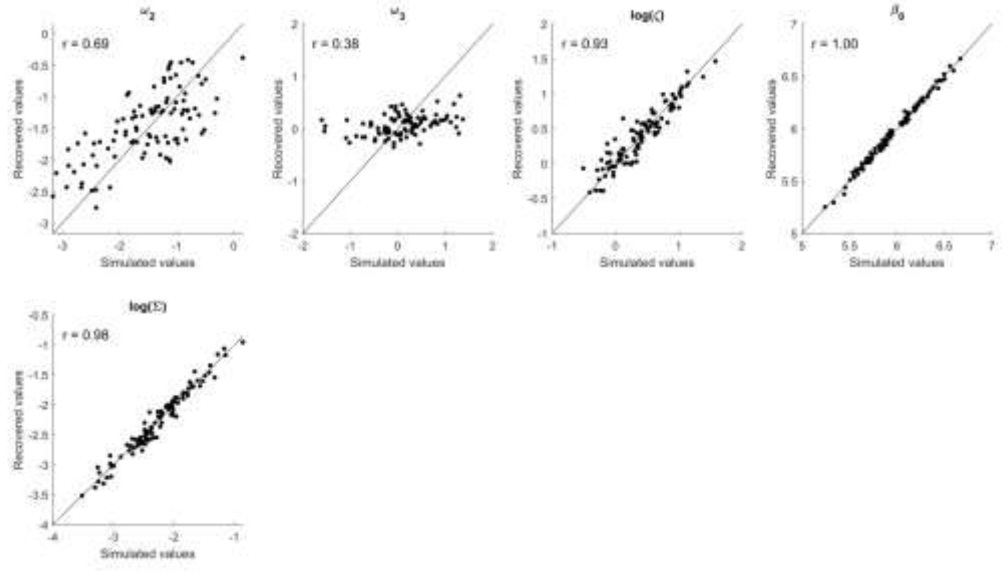

Figure S3D | Parameter recovery of M5. $N_{sim} = 100$ parameter values are displayed (simulated parameter values on the x-axis, fitted parameter values on the y-axis) and Pearson correlation coefficients between simulated and estimated parameter values for each free parameter

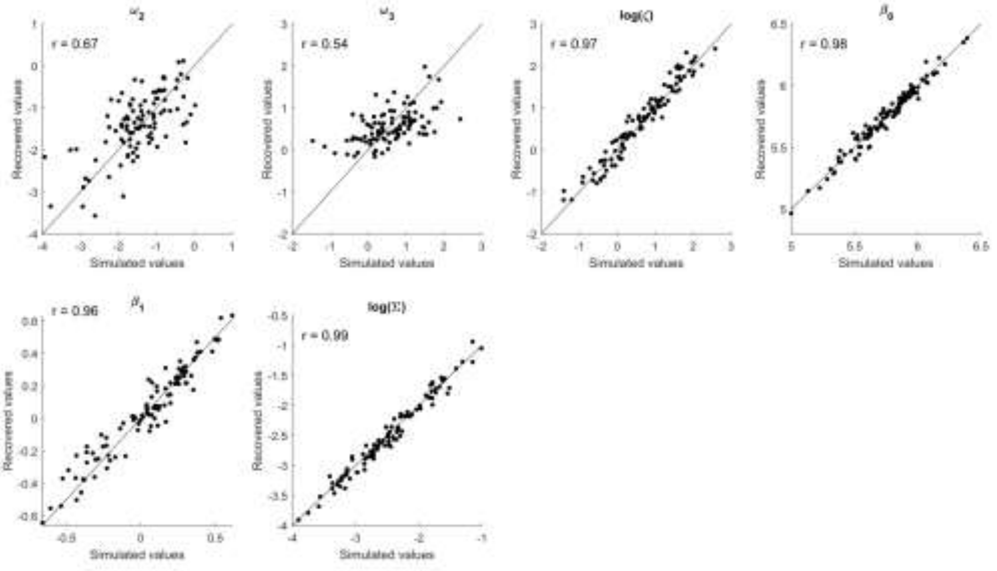

Figure S3E | Parameter recovery of M6. $N_{sim} = 100$ parameter values are displayed (simulated parameter values on the x-axis, fitted parameter values on the y-axis) and Pearson correlation coefficients between simulated and estimated parameter values for each free parameter

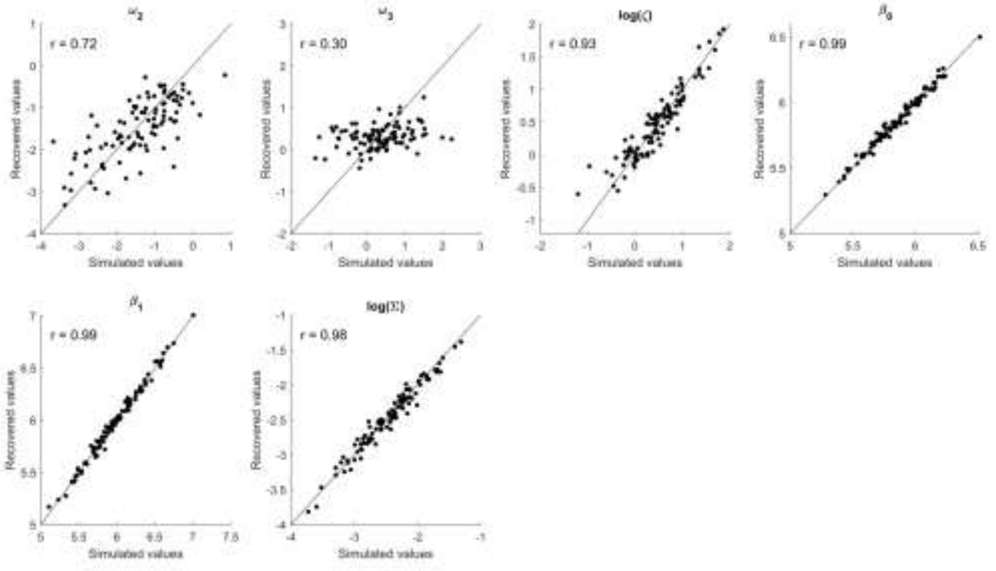

Figure S3F | Parameter recovery of M7. $N_{sim} = 100$ parameter values are displayed (simulated parameter values on the x-axis, fitted parameter values on the y-axis) and Pearson correlation coefficients between simulated and estimated parameter values for each free parameter

S4: Average log RT trajectories and log RT model fits of M1-M7

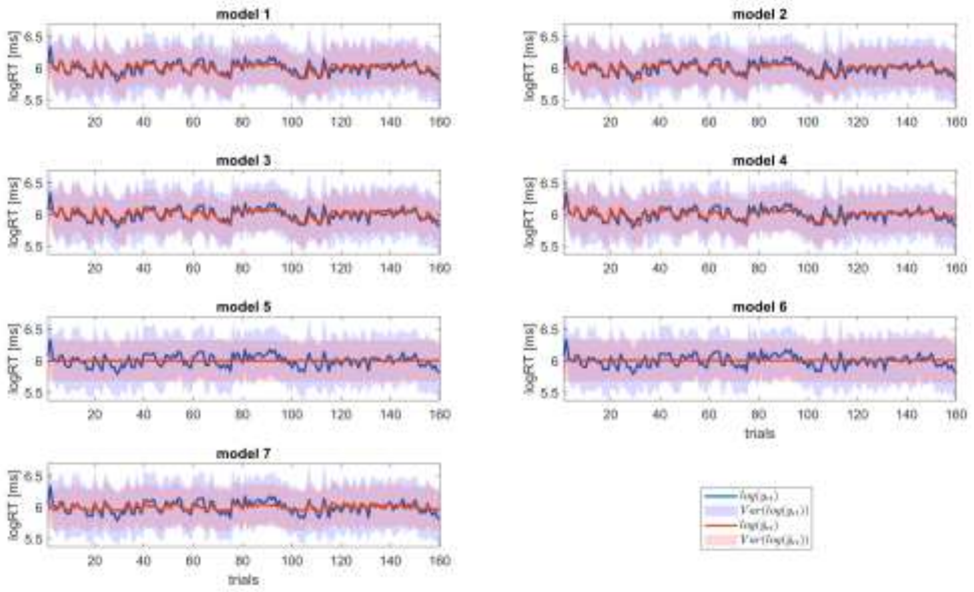

Figure S4 | Average of the predicted (M1-M7) and average of the actual log RTs. In blue, mean and variance of log RT trajectories over all participants of the main data set are displayed ($N_{main} = 59$). In red, mean and variance of the log RT model fits is shown for each of the seven models in the model space.

S5: Posterior predictive checks for M1

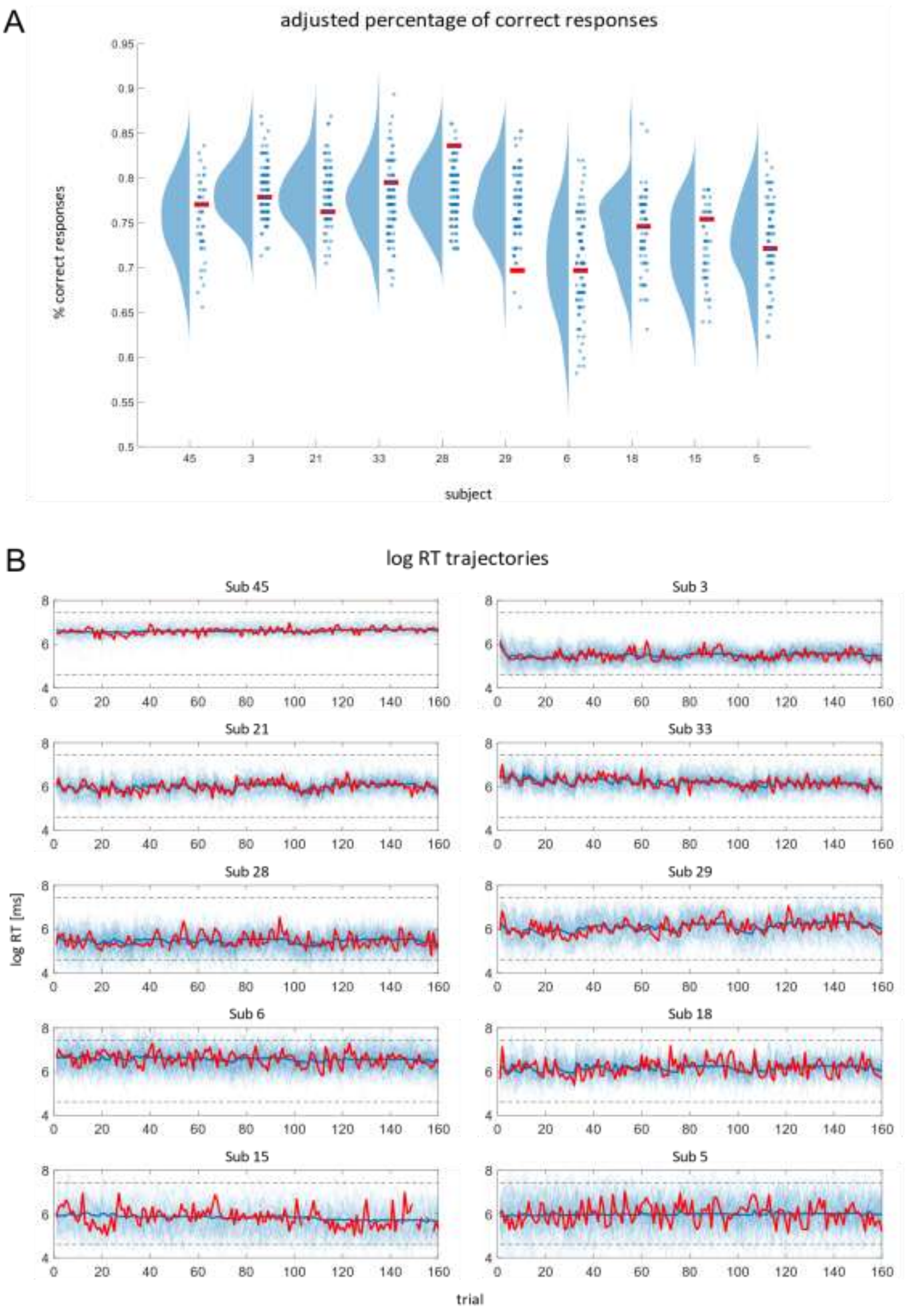

Figure S5 | Posterior predictive checks for M1. Data from ten participants of the main data set are shown. Participants were chosen according to goodness of model fit of M1, i.e. the four

participants with the highest log likelihood values (45, 3, 21, 33), two participants with average goodness of fit (28, 29) and the four participants showing the worst fit (6, 18, 15, 5). **A** displays adjusted correctness of binary responses for these participants in red. Blue circles are the simulated adjusted correctness values resulting from sampled parameter values of the subject-specific posteriors of M1. The blue probability densities are the estimated posterior predictive densities based on the samples drawn from the posteriors ($N_{ppc} = 100$) using kernel density estimation as implemented in the RainCloudPlots library. In **B**, we show empirical log RT trajectories of the ten participants in red. Fine blue lines are simulated log RT trajectories resulting from sampled parameter values of the subject-specific posteriors and the thick blue line represents the predicted log RT when using the MAP estimates of M1 for each participant to generate synthetic RT data.

S6: Repeated Hypothesis testing using only six models (M2-M7)

One of our reviewers understandably wondered why M1 was clearly outperforming M3 in the RFX BMS (Figure 5B), given that the two models share one regressor (the outcome uncertainty $\hat{\sigma}_1^{(k)}$), whose associated regression weight was significantly different from zero in (Figure 7C). Although this result may be counterintuitive at first glance, it has a simple explanation:

First, the fact that Bayesian model comparison demonstrates a major difference in how well M1 and M3 explain the data suggests that those regressors of M1 that are not contained in M3 improve LME estimates at the single-subject level (i.e. increase accuracy more than they increase complexity) and do so consistently across subjects. Indeed, this is shown in Figure S6A which plots the LME difference between M1 and M3 for all subjects individually. Notably, this beneficial effect of the regressors in M1 on the LME estimate does not depend on whether the sign of the associated regression coefficients is consistent across participants are not. It is therefore not expected that the regression weights of those regressors in M1 that are not contained by M3 are necessarily significant (compare Figure 7). This is not the first time such a situation is encountered; we refer the interested reader to a previous result by Piray et al., (2017) who described a similar case, albeit in a different context (RFX BMS of dynamic causal models of fMRI data).

Second, it is worth keeping in mind that RFX BMS results are conditional on the model space considered. In other words, the relative performance of a given model within a set of candidate models can change if the model space is altered. Such effects are typically particularly visible when adding or removing a model that is similar (in terms of its parameterisation) to another model in the model space. It is therefore possible that the surprisingly poor performance of M3, as shown in Figure 7, might arise because it (i) shows some degree of similarity to M1 but (ii) is consistently outperformed by M1 across subjects (as shown in Figure S6A). To confirm this intuition, we repeated the RFX BMS analysis on the same data set using only M2-M7 and show the results in Supplementary Figure S6 (parts B and C). Supplementary Figure S6C shows that it is now indeed M3 that outperforms all other models (in the absence of M1). (For completeness, is worth mentioning that, at the same time, M2-M4 still outperform M5-M7 at the family level as shown in Supplementary Figure S6B.)

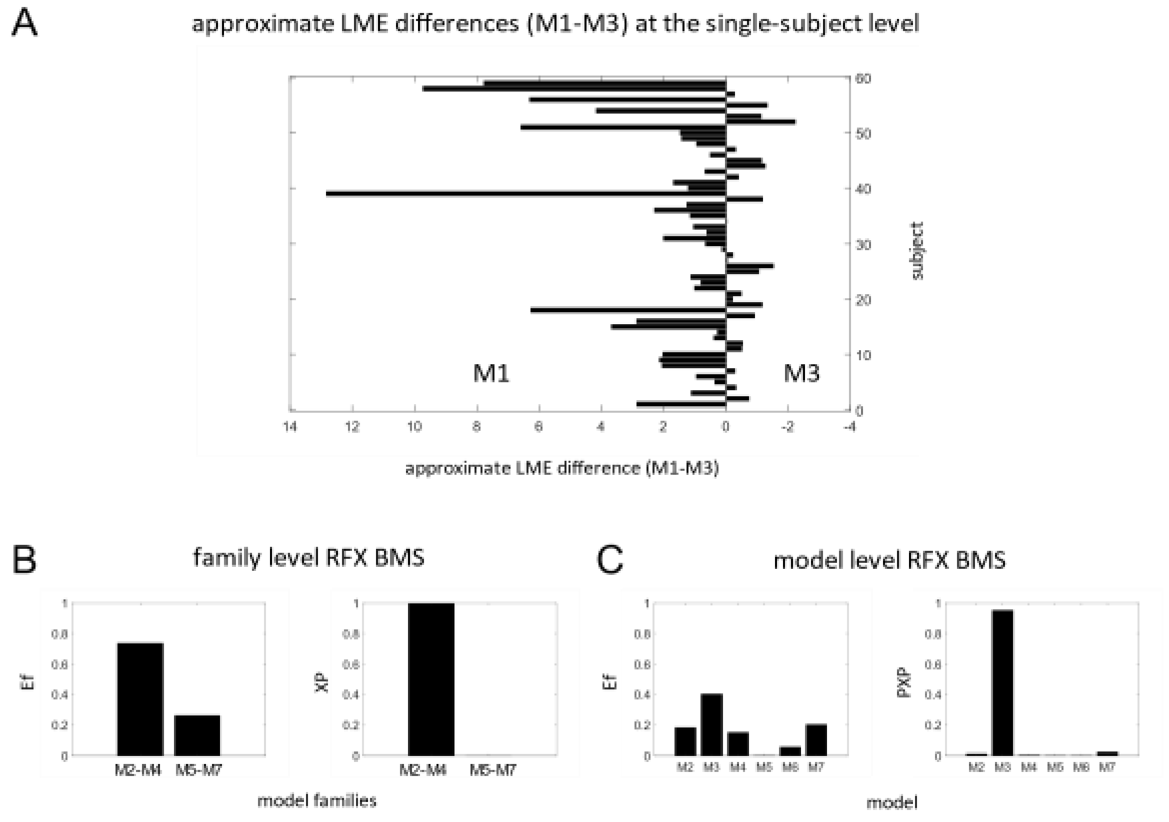

Figure S6 | Single-subject approximate LME differences and repeated Hypothesis testing using only six models (M2-M7). **A** shows differences in approximate LME values between M1 and M3 at the single-subject level. **B** shows the results of the family level RFX BMS (Efs on the left, XPs on the right) with M2-M4 clearly outperforming the uninformed RT model family (M5-M7) despite the absence of M1. **C** displays Efs (left) and PXPs (right) resulting from individual model level RFX BMS. In the absence of M1, M3 can be identified as the clear winning model.

References

Piray, P., den Ouden, H.E.M., van der Schaaf, M.E., Toni, I., Cools, R., 2017. Dopaminergic Modulation of the Functional Ventrodorsal Architecture of the Human Striatum. Cereb. Cortex 27, 485–495. https://doi.org/10.1093/cercor/bhv243
